# Association of body mass index with outcomes in patients with newly diagnosed atrial fibrillation: GARFIELD-AF

**DOI:** 10.1136/openhrt-2022-002038

**Published:** 2022-08-05

**Authors:** Christian Fielder Camm, Saverio Virdone, Shinya Goto, Jean-Pierre Bassand, Martin van Eickels, Sylvia Haas, Bernard J Gersh, Karen Pieper, Keith A A Fox, Frank Misselwitz, Alexander G G Turpie, Samuel Z Goldhaber, Freek Verheugt, John Camm, Gloria Kayani, Elizaveta Panchenko, Seil Oh, Hector Lucas Luciardi, Jitendra Pal Singh Sawhney, Stuart J Connolly, Pantep Angchaisuksiri, Hugo ten Cate, John W Eikelboom, Ajay K Kakkar, the GARFIELD-AF investigators, Ajay K. Kakkar

**Affiliations:** 1 Keble College, University of Oxford, Oxford, UK; 2 Cardiology Department, Royal Berkshire NHS Foundation Trust, Reading, UK; 3 Thrombosis Research Institute, London, UK; 4 School of Medicine Graduate School of Medicine, Tokai University, Isehara, Japan; 5 Universite de Besancon, Besancon, France; 6 Bayer AG, Leverkusen, Germany; 7 Haemostasis and Thrombosis Research Group, Institute for Experimental Oncology and Therapy Research, Technical University of Munich, Munich, Germany; 8 Mayo Clinic, Rochester, Minnesota, USA; 9 The University of Edinburgh School of Clinical Sciences, Edinburgh, UK; 10 Royal Infirmary of Edinburgh, Edinburgh, UK; 11 Bayer AG, Berlin, Germany; 12 Department of Medicine, McMaster University, Hamilton, Ontario, Canada; 13 Department of Medicine, Brigham and Women's Hospital, Boston, Massachusetts, USA; 14 Department of Cardiology, Onze Lieve Vrouwe Gasthuis, Amsterdam, The Netherlands; 15 Department of Cardiology, St George's University of London, London, UK; 16 Ministry of Health of the Russian Federation, Moskva, Russian Federation; 17 Department of Internal Medicine, Seoul National University Hospital, Seoul, Korea (the Republic of); 18 National University of Tucuman, San Miguel de Tucuman, Tucumán, Argentina; 19 Sir Ganga Ram Hospital, Lahore, Pakistan; 20 McMaster University, Hamilton, Ontario, Canada; 21 Department of Medicine, Mahidol University, Salaya, Thailand; 22 Maastricht University Cardiovascular Research Institute Maastricht, Maastricht, The Netherlands; 23 Maastricht University Medical Centre+, Maastricht, The Netherlands; 24 Department of Surgery, University College London, London, UK

**Keywords:** Atrial Fibrillation, Obesity, Epidemiology

## Abstract

**Objective:**

While greater body mass index (BMI) is associated with increased risk of developing atrial fibrillation (AF), the impact of BMI on outcomes in newly diagnosed AF is unclear. We examine the influence of BMI on outcomes and whether this is modified by sex and evaluate the effect of non-vitamin K oral anticoagulants (NOACs) in patients with high BMI.

**Methods:**

GARFIELD-AF is a prospective registry of 52 057 newly diagnosed AF patients. The study population comprised 40 482 participants: 703 underweight (BMI <18.5 kg/m^2^), 13 095 normal (BMI=18.5–24.9 kg/m^2^), 15 043 overweight (BMI=25.0–29.9 kg/m^2^), 7560 obese (BMI=30.0–34.9 kg/m^2^) and 4081 extremely obese (BMI ≥35.0 kg/m^2^). Restricted cubic splines quantified the association of BMI with outcomes. Comparative effectiveness of NOACs and vitamin K antagonists (VKAs) by BMI was performed using propensity score overlap-weighted Cox models.

**Results:**

The median age of participants was 71.0 years (Q1; Q3 62.0; 78.0), and 55.6% were male. Those with high BMI were younger, more often had vascular disease, hypertension and diabetes. Within 2-year follow-up, a U-shaped relationship between BMI and all-cause mortality was observed, with BMI of ~30 kg/m^2^ associated with the lowest risk. The association with new/worsening heart failure was similar. Only low BMI was associated with major bleeding and no association emerged for non-haemorrhagic stroke. BMI was similarly associated with outcomes in men and women. BMI did not impact the lower rate of all-cause mortality of NOACs compared with VKAs.

**Conclusions:**

In the GARFIELD-AF registry, underweight and extremely obese AF patients have increased risk of mortality and new/worsening heart failure compared with normal or obese patients.

**WHAT IS ALREADY KNOWN ON THIS TOPIC**

- Evidence supports an association between high body mass index and risk of atrial fibrillation (AF). However, the evidence of an association between BMI and AF-related outcomes remains unclear.

**WHAT THIS STUDY ADDS**

- A U-shaped association between BMI and all-cause mortality and heart failure in AF patients is revealed, indicating that underweight or extremely obese patients have a significantly higher risk of mortality and heart failure in comparison with those with normal body weight, overweight or obese patients. BMI, however, does not significantly impact the benefits of non-vitamin K oral anticoagulants (NOACs) versus vitamin K antagonists therapy.

**HOW THIS STUDY MIGHT AFFECT RESEARCH, PRACTICE OR POLICY**

- In clinical practise, BMI should be measured and considered as a risk factor for outcomes in AF. NOACs could be considered for the treatment of all patients regardless of associated BMI.

## Introduction

Atrial fibrillation (AF) is a common arrhythmia associated with significant risk of mortality, cardioembolic stroke and heart failure.^1^ Evidence supports an association between higher body mass index (BMI) and risk of AF as well as other anthropometric measures including waist circumference, weight and height.^2^ The association of higher BMI with risk of AF-related outcomes is unclear, with some studies suggesting an inverse relationship.^3 4^ As obesity is generally considered detrimental to health, this inverse relationship has been described as an ‘obesity paradox’.^5^ However, this finding has not been consistent.^6^

Significant sex-based differences exist for both the incidence of AF and the risk of AF-related outcomes.^7^ Additionally, anthropometric measures differ between men and women.^8^ In particular, body fat distribution differs substantially.^2 9^ At present, it is unclear whether the association between anthropometric measures and the risk of AF-related outcomes is similar in men and women.

Given the higher risk of non-haemorrhagic stroke in patients with AF, treatment with oral anticoagulation is often recommended.^10^ However, concerns have been raised regarding the safety and efficacy of non-vitamin K oral anticoagulants (NOACs) in obese and extremely obese populations.^11^

This study evaluates a large international cohort of participants with new-onset AF in GARFIELD-AF. The three principal aims were to: (1) assess associations between BMI, weight and height and the risk of AF-associated outcomes; (2) explore sex-based differences in these associations; and (3) examine the association of obesity with the impact of NOAC therapy in patients with AF.

## Methods

### Study design and participants

The GARFIELD-AF design has been previously published (ClinicalTrials.gov identifier: NCT01090362).^12 13^ Eligible participants (≥18 years) required a recent diagnosis of new onset non-valvular AF and ≥1 further stroke risk factor. Patients (n=52 057) were enrolled prospectively and consecutively from 35 countries globally into five cohorts from 2010 to 2016, without exclusions according to treatment or comorbidities. Study sites were computationally selected at random from a list of representative care settings. Treatment and therapy decisions were at the discretion of the physician and patient.

Patients <20 years of age, without BMI data or for whom follow-up information was unavailable were omitted from the analysis. Those with a BMI of <15 kg/m^2^ or >60 kg/m^2^ were excluded due to likely measurement error in the calculation of height or weight.

### Data collection

Data were collected using electronic case report forms that were securely submitted electronically to the registry-coordinating centre Dendrite Clinical Systems Ltd (Henley-on-Thames, UK). Accuracy and completeness of data was ensured by the coordinating centre, the Thrombosis Research Institute (London, UK). Data were extracted from the study data base on 30 June 2019.

### Ethics

The GARFIELD-AF registry is conducted in accordance with the principles of the Declaration of Helsinki, local regulatory requirements and International Conference on Harmonization–Good Pharmacoepidemiological and Clinical Practice guidelines.

### Patient and public involvement statement

Written informed consent was obtained from all study participants. There was no public or patient involvement in the design or execution of the GARFIELD-AF study design. Investigators from representative investigator sites in each participating country were involved in data collection. Confidentiality and anonymity were maintained through assigned unique identifiers. Independent ethics committee and hospital-based institutional review board approvals were obtained. A full list of ethics committees is provided in the online supplemental material 1.

10.1136/openhrt-2022-002038.supp1 Supplementary data

### BMI categories

Patients were categorised into five BMI groups according to the WHO definitions: underweight (<18.5 kg/m^2^), normal (18.5–24.9 kg/m^2^), overweight (25.0–29.9 kg/m^2^), obese (30–34.9 kg/m^2^) and extremely obese (≥35.0 kg/m^2^).^14^

### Clinical characteristics and outcomes

Demographic and clinical characteristics were recorded at baseline. The primary clinical outcomes included the rate of mortality, non-haemorrhagic stroke/systemic embolism (SE), major bleeding and new/worsening heart failure according to BMI. These outcomes were also analysed according to BMI and sex. Major bleeding was defined as clinically overt bleeding associated with a critical site, a fall in haemoglobin (≥2 g/dL), transfusion of packed red blood cells (≥2 units), haemorrhagic stroke or fatal outcome.

### Statistical analysis

Continuous variables are reported using medians and IQR. Categorical variables are presented as percentages and frequency counts. Data for the CHA_2_DS_2_-VASc and HAS-BLED risk scores were collected; the latter was calculated excluding labileinternational normalized ratios (INRs) as this was not recorded at baseline. Clinical outcomes are described by number of events, event rate per 100 person-years estimated via a Poisson model and 95% CIs. Only the first occurrence of each event was considered. Survival differences across BMI groups were examined using Kaplan-Meier curves.

To identify non-linear association between the continuous variables BMI, weight and height with outcome events, analyses of restricted cubic splines were performed by generating restricted cubic splines with four knots and applying a separate Cox multivariable model for each studied endpoint.

Covariate adjustments for Cox models were selected by avoiding the inclusion of factors in the mediating pathway between BMI and clinical outcomes. Apart from the selected demographic characteristic, smoking status and alcohol consumption were included because of their established relationship with BMI. Chronic kidney disease (CKD) was also considered a possible confounder, as CKD often causes weight loss in later stages.^15 16^

Three sensitivity analyses were performed; the first analysed the rate of new/worsening heart failure in patients without baseline heart failure. The second examined the association of BMI and AF outcomes in patients with ≥6 months of follow-up. The third adjusted associations for a broader range of covariates.

Comparative effectiveness analyses of NOAC versus VKA were performed in a study population restricted to anticoagulated patients at baseline with a CHA_2_DS_2_-VASc score ≥2 (excluding sex) enrolled in GARFIELD-AF cohorts 3–5 (NOACs were not widely available for patients enrolled in earlier cohorts). Analyses were performed for patients with a normal/overweight BMI (18.5–29.9 kg/m^2^) and separately for those with an obese/extremely obese BMI (≥30 kg/m^2^). Patients with a BMI <18.5 were not included. HRs for NOAC versus VKA were obtained using a Cox proportional hazards model via a propensity method of overlap weighting to balance covariates in the population. Treatment was defined as the first treatment received at the time of enrolment, approximating ‘intention-to-treat’. Absolute standardised differences for NOAC versus VKA comparison before and after propensity score weighting among normal weight or overweight patients (BMI 18.5–29.9 kg/m^2^) and obese or extremely obese patients (BMI ≥30 kg/m^2^) have been presented in online supplemental figure S1a and S1b, respectively.

**Figure 1:**
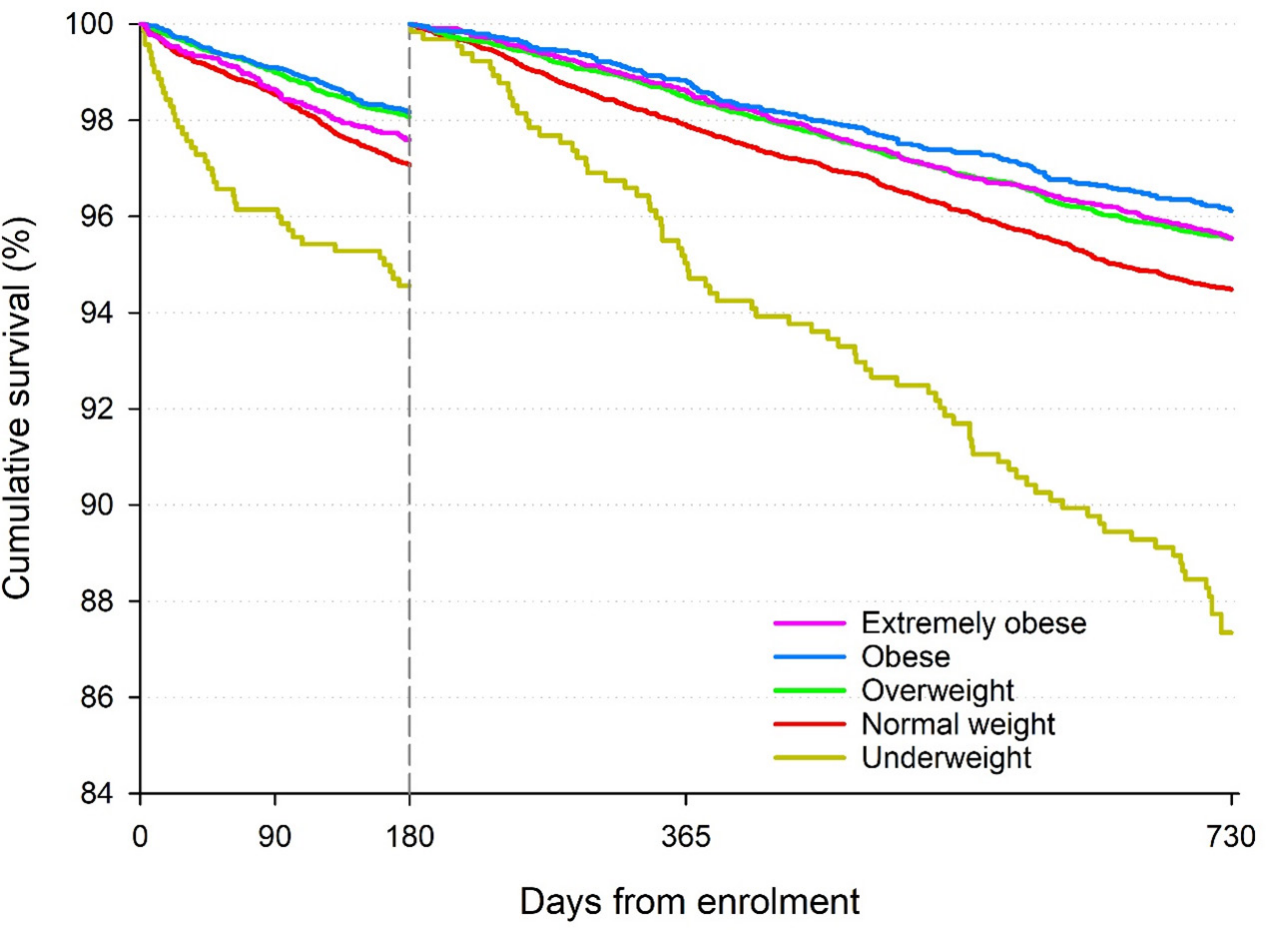
Cumulative survival at 6 months and conditional at having survived 6 months by BMI category. BMI, body mass index.

Only complete cases are presented in descriptive tables. Multiple imputation was applied in the derivation for the modelling process for the estimation of the BMI effect; coefficients and SEs for the risk models were obtained by combining estimates across five imputed datasets. Single imputation was applied for the NOAC versus VKA comparison. P<0.05 were considered statistically significant. Statistical analyses were carried out using SAS (V.9.4).

## Results

### Baseline characteristics

A total of 40 482 participants were eligible for inclusion within the main analysis: underweight (n=703), normal weight (n=13 095), overweight (15,043), obese (n=7560) and extremely obese (n=4081). The median age of participants was 71.0 years (Q1; Q3: 62.0; 78.0) and slightly over half of the participants were male (n=22 881, 56.5%). Median BMI was 26.9 kg/m2 (Q1; Q3: 24.0; 30.7 kg/m^2^), and median weight was 75.0 kg (Q1; Q3: 65.0; 88.0 kg). Those with higher BMI were generally younger, more often had diabetes mellitus, hypertension and vascular disease at enrolment and were more likely to have been diagnosed with permanent AF. Baseline demographic characteristics are provided intable 1). Baseline anticoagulation patterns differed significantly among BMI categories; underweight patients were less likely to receive anticoagulation treatment at baseline compared with other groups (60.3% vs 67.9%, p<0.0001, table 1), even when considering only patients at high risk of stroke (ie, CHA_2_DS_2_-VASc≥2, 62.1% vs 70.1%, p<0.0001, online supplemental figure S2). Medical history at baseline is provided in table 2.

**Figure 2:**
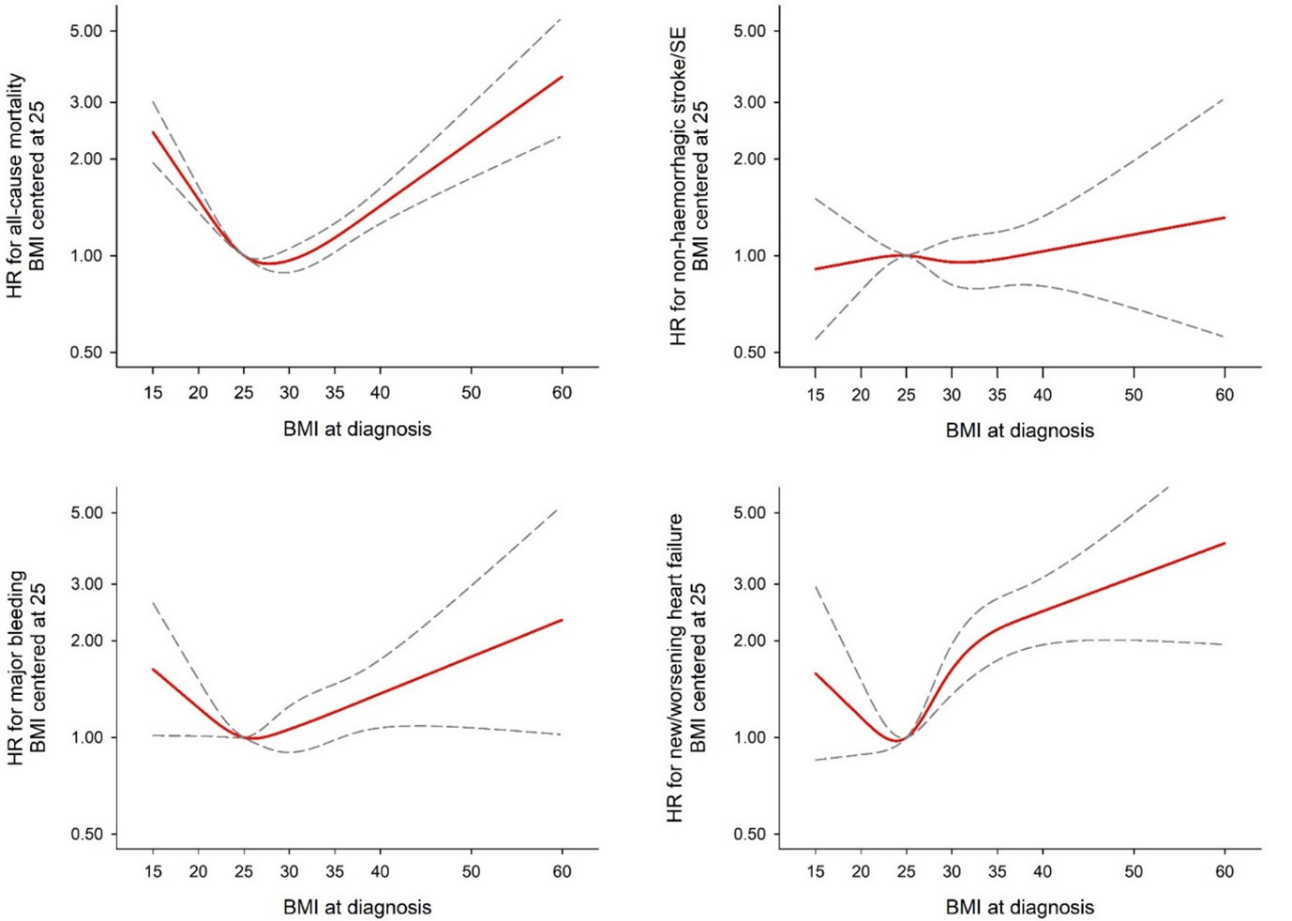
Adjusted* associations through 2-year follow-up between BMI and selected endpoints based on a restricted cubic spline model. *Adjusted by age, sex, ethnicity, smoking status, alcohol use and moderate to severe CKD. BMI, body mass index; CKD, chronic kidney disease.

**Table 1:** Baseline characteristics by BMI category

Baseline characteristics	BMI category				
	Underweight (n=703)	Normal weight (n=13 095)	Overweight (n=15 043)	Obese (n=7560)	Extremely obese (n=4081)
Male, n (%)	275 (39.1)	7228 (55.2)	9276 (61.7)	4195 (55.5)	1907 (46.7)
Age, median (Q1; Q3), years	77.0 (68.0–84.0)	73.0 (64.0–80.0)	71.0 (62.0–78.0)	69.0 (61.0–76.0)	66.0 (59.0–73.0)
Age, n (%), years					
<65	117 (16.6)	3485 (26.6)	4583 (30.5)	2599 (34.4)	1823 (44.7)
65–69	88 (12.5)	1795 (13.7)	2354 (15.6)	1314 (17.4)	751 (18.4)
70–74	95 (13.5)	2215 (16.9)	2638 (17.5)	1340 (17.7)	636 (15.6)
≥75	403 (57.3)	5600 (42.8)	5468 (36.3)	2307 (30.5)	871 (21.3)
Ethnicity, n (%)					
Caucasian	204 (29.5)	5710 (44.3)	9684 (65.4)	5786 (78.0)	3309 (82.6)
Hispanic/Latino	20 (2.9)	654 (5.1)	1042 (7.0)	628 (8.5)	330 (8.3)
Asian	463 (66.9)	6353 (49.2)	3783 (25.6)	791 (10.7)	196 (4.9)
Afro-Caribbean/mixed/other	5 (0.7)	185 (1.4)	297 (2.0)	210 (2.8)	163 (4.1)
Weight, median (Q1–Q3), kg	45.0 (40.0–49.0)	62.0 (55.0–69.0)	77.0 (70.0–84.0)	90.0 (82.0–98.0)	108.0 (96.0–120.0)
Height, median (Q1–Q3), cm	160.0 (154.0–168.0)	165.0 (158.0–172.0)	168.0 (161.0–175.0)	168.0 (160.0–175.0)	166.0 (159.0–174.0)
SBP, median (Q1–Q3), mm Hg	126.0 (112.0–140.0)	130.0 (119.0–140.0)	131.0 (120.0–144.0)	135.0 (121.0–148.0)	136.0 (123.0–149.0)
DBP, median (Q1–Q3), mm Hg	74.0 (66.0–80.0)	79.0 (70.0–85.0)	80.0 (70.0–88.0)	80.0 (73.0–90.0)	80.0 (74.5–90.0)
Pulse, median (Q1–Q3), bpm	85.0 (70.0–103.0)	81.0 (70.0–100.0)	81.0 (70.0–100.0)	84.0 (70.0–104.0)	88.0 (73.0–110.0)
Type of AF, n (%)					
Permanent	66 (9.4)	1610 (12.3)	2027 (13.5)	1050 (13.9)	584 (14.3)
Persistent	127 (18.1)	1979 (15.1)	2454 (16.3)	1232 (16.3)	686 (16.8)
Paroxysmal	260 (37.0)	4378 (33.4)	4210 (28.0)	1884 (24.9)	839 (20.6)
New onset (unclassified)	250 (35.6)	5128 (39.2)	6352 (42.2)	3394 (44.9)	1971 (48.3)
Specialty at diagnosis, n (%)					
Internal medicine/neurology/geriatrics	144 (20.5)	2404 (18.4)	2958 (19.7)	1520 (20.1)	891 (21.8)
Cardiology	503 (71.6)	9419 (71.9)	10 017 (66.6)	4793 (63.4)	2446 (60.0)
Primary care/general practice	56 (8.0)	1272 (9.7)	2068 (13.7)	1247 (16.5)	743 (18.2)
Care setting at diagnosis, n (%)					
Hospital	439 (62.4)	8039 (61.4)	8806 (58.5)	4106 (54.3)	2215 (54.3)
Office/anticoagulation clinic/thrombosis centre	224 (31.9)	4021 (30.7)	4799 (31.9)	2550 (33.7)	
Emergency room	40 (5.7)	1035 (7.9)	1438 (9.6)	904 (12.0)	479 (11.7)
Heavy alcohol use, n (%)	12 (1.9)	287 (2.5)	303 (2.3)	151 (2.2)	64 (1.8)
Current smoker, n (%)	79 (11.9)	1542 (12.6)	1568 (11.1)	694 (9.7)	380 (9.8)
Treatment, n (%)					
NOAC±AP	185 (26.5)	3739 (28.9)	4137 (27.9)	2048 (27.6)	1184.6)
VKA±AP	235 (33.7)	4502 (34.8)	5952 (40.2)	3265 (44.0)	1770.3)
AP only	146 (20.9)	2906 (22.5)	3190 (21.5)	1466 (19.7)	675 (16.9)
None	131 (18.8)	1787 (13.8)	1545 (10.4)	644 (8.7)	367 (9.2)

AF, atrial fibrillation; AP, antiplatelet; BMI, body mass index; DBP, diastolic blood pressure; NOAC, non-oral anticoagulant; SBP, systolic blood pressure; VKA, vitamin K antagonist.

**Table 2:** Medical history by BMI category

Baseline comorbidity or risk score/tool, n (%)	BMI category				
	Underweight (n=703)	Normal weight (n=13 095)	Overweight (n=15 043)	Obese (n=7560)	Extremely obese (n=4081)
Heart failure	203 (28.9)	2972 (22.7)	3281 (21.8)	1969 (26.0)	1221 (29.9)
Acute coronary syndromes	64 (9.1)	1269 (9.7)	1706 (11.4)	935 (12.4)	429 (10.6)
Vascular disease*	147 (21.0)	2960 (22.8)	3877 (25.9)	2182 (29.1)	1106 (27.3)
Carotid occlusive disease	22 (3.2)	397 (3.1)	525 (3.5)	219 (2.9)	87 (2.2)
VTE	9 (1.3)	213 (1.6)	362 (2.4)	245 (3.3)	200 (4.9)
Prior stroke/TIA/SE	101 (14.5)	1575 (12.1)	1693 (11.3)	756 (10.1)	356 (8.8)
Prior bleeding	28 (4.0)	326 (2.5)	384 (2.6)	197 (2.6)	107 (2.6)
Hypertension	388 (55.3)	8945 (68.5)	11 816 (78.7)	6490 (86.0)	3603 (88.5)
Hypercholesterolaemia	157 (23.1)	4216 (33.0)	6481 (44.3)	3821 (52.3)	2088 (52.7)
Diabetes	69 (9.8)	2099 (16.0)	3190 (21.2)	2238 (29.6)	1590 (39.0)
Cirrhosis	7 (1.0)	83 (0.6)	81 (0.5)	50 (0.7)	28 (0.7)
Moderate to severe CKD	95 (14.1)	1455 (11.4)	1456 (10.0)	783 (10.7)	449 (11.4)
Dementia	31 (4.4)	265 (2.0)	169 (1.1)	71 (0.9)	28 (0.7)
CHA_2_DS_2_-VASc score, median (Q1–Q3)	4.0 (2.0–4.0)	3.0 (2.0–4.0)	3.0 (2.0–4.0)	3.0 (2.0–4.0)	3.0 (2.0–4.0)
HAS-BLED score†, median (Q1–Q3)	1.0 (1.0–2.0)	1.0 (1.0–2.0)	1.0 (1.0–2.0)	1.0 (1.0–2.0)	1.0 (1.0–2.0)
GARFIELD-AF death score‡, median (Q1–Q3)	9.1 (4.8–16.9)	5.2 (2.9–10.1)	4.4 (2.5–8.1)	4.5 (2.6–7.6)	4.1 (2.4–6.9)
GARFIELD-AF stroke score§, median (Q1–Q3)	2.1 (1.4–3.0)	1.7 (1.1–2.5)	1.5 (1.1–2.3)	1.5 (1.0–2.2)	1.4 (0.9–2.1)
GARFIELD-AF bleeding score¶, median (Q1–Q3)	1.9 (1.2–3.1)	1.6 (1.0–2.5)	1.6 (1.0–2.4)	1.5 (1.0–2.3)	1.4 (0.9–2.1)

*Defined as peripheral artery disease and/or coronary artery disease.

†The risk factor ‘Labile INRs’ is not included in the HAS-BLED score as it is not collected at baseline. As a result, the maximum HAS-BLED score at baseline is 8 points (not 9).

‡Represent the risk of mortality within 2 years.

§Represent the risk of non-haemorrhagic stroke within 2 years.

¶Represent the risk of major bleeding within 2 years.

CKD, chronic kidney disease; SE, systemic embolism; TIA, transient ischaemic attack; VTE, venous thromboembolism.

### Events

During a 2-year follow-up, 2805 participants (6.9%) died, 770 (1.9%) suffered a non-haemorrhagic stroke/SE, 730 (1.8%) had a major bleeding event and 589 (1.5%) had a new or worsening heart failure event (online supplemental table S1). Cumulative survival at 6 months, and past 6 month, according to BMI category is shown in figure 1.

### BMI association with outcomes

After adjustment, restricted cubic spline regression demonstrated a U-shaped relationship between BMI and all-cause mortality and new/worsening heart failure; both low and high BMI were positively associated with events (figure 2). The lowest risk of all-cause mortality was observed at a BMI of ~30 kg/m^2^. Below 30 kg/m^2^, there was a 32% higher risk of mortality per 5 kg/m^2^ lower BMI (95% CI 25% to 40%). Above 30 kg/m^2^, there was a 16% higher risk of mortality per 5 kg/m^2^ higher BMI (95% CI 9% to 23%). The lowest risk of new/worsening heart failure was at ~25 kg/m^2^. Above 25 kg/m^2^, each 5 kg/m^2^ higher BMI was associated with a 23% higher risk (95% CI 14% to 33%). For major bleeding, there was a positive association only in those with low BMI. There was no association between non-haemorrhagic stroke and BMI.

### Association of height and weight with outcomes

**Figure 3:**
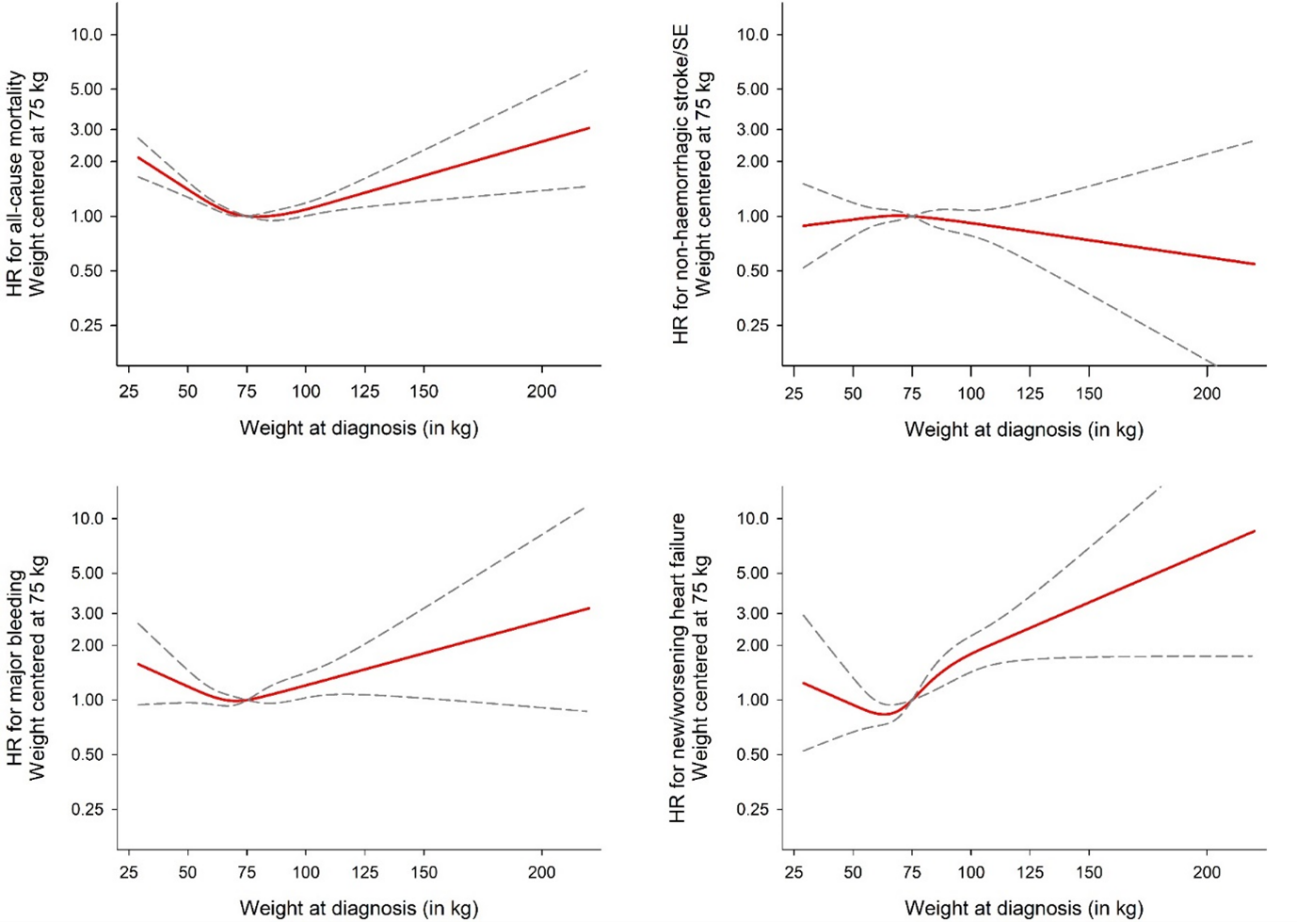
Adjusted* associations through 2-year follow-up between weight and selected endpoints based on a restricted cubic spline model. *Adjusted by age, sex, ethnicity, smoking status, alcohol use and moderate to severe CKD. CKD, chronic kidney disease.

**Figure 4:**
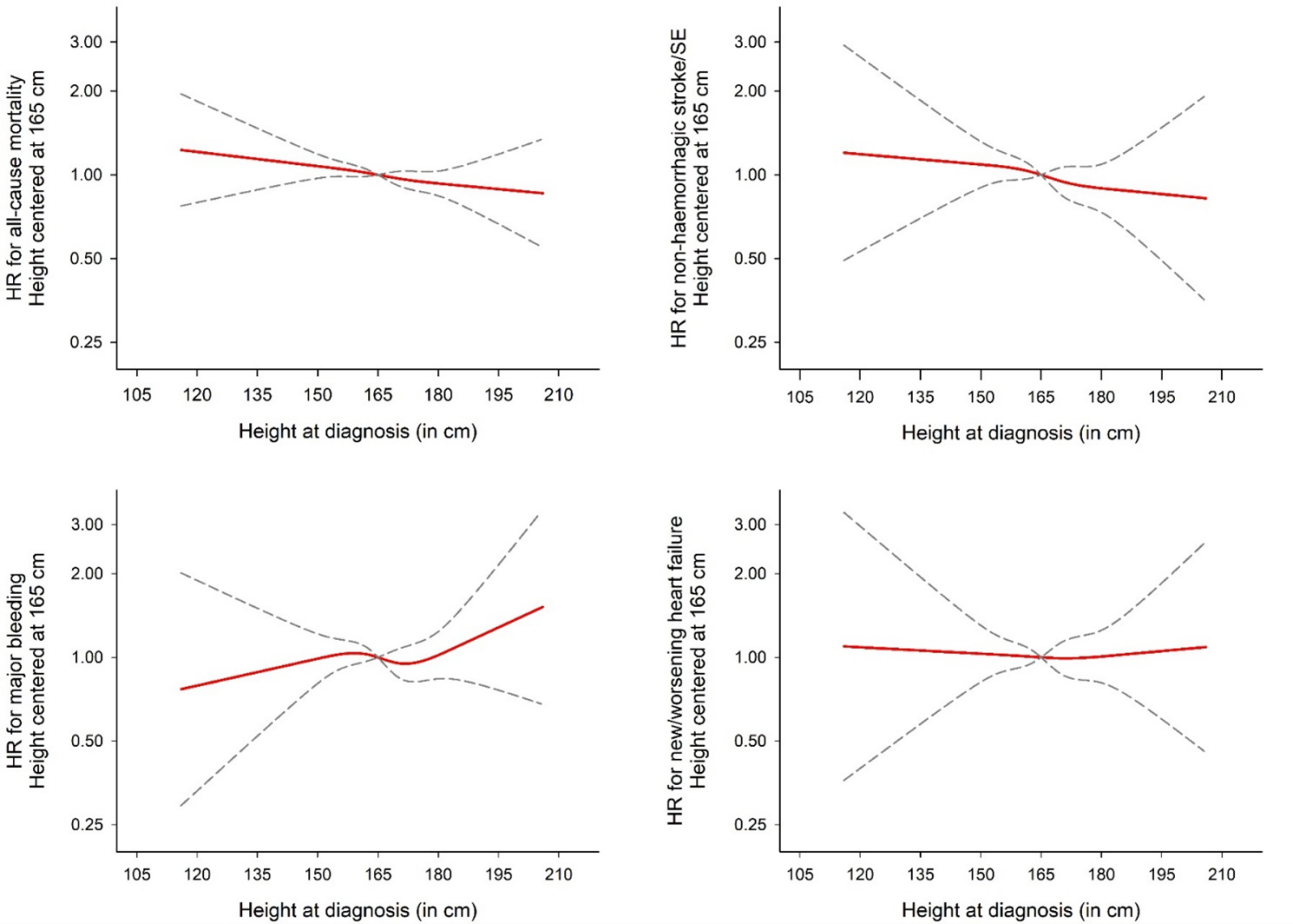
Adjusted* associations through 2-year follow-up between height and selected endpoints based on a restricted cubic spline model.*Adjusted by age, sex, ethnicity, smoking status, alcohol use, and moderate to severe CKD.

As with BMI, weight showed similar associations with AF outcomes. A U-shaped relationship was observed between weight and all-cause mortality and new/worsening heart failure, with both low and high weight being positively associated (figure 3). The lowest risk for both outcomes was at ~75 kg. Below 75 kg, there was a 15% higher risk of all-cause mortality per 5 kg lower weight (95% CI 12% to 18%). For every 5 kg higher weight above 75 kg, there was a 1% higher risk of all-cause mortality (95% CI 0% to 3%) and 6% higher risk of new/worsening heart failure (95% CI 3% to 9%). No significant association was observed between weight and the risk of major bleeding or non-haemorrhagic stroke. There was no association between height and AF outcomes (figure 4).

### Association between BMI and AF outcomes by sex

**Figure 5:**
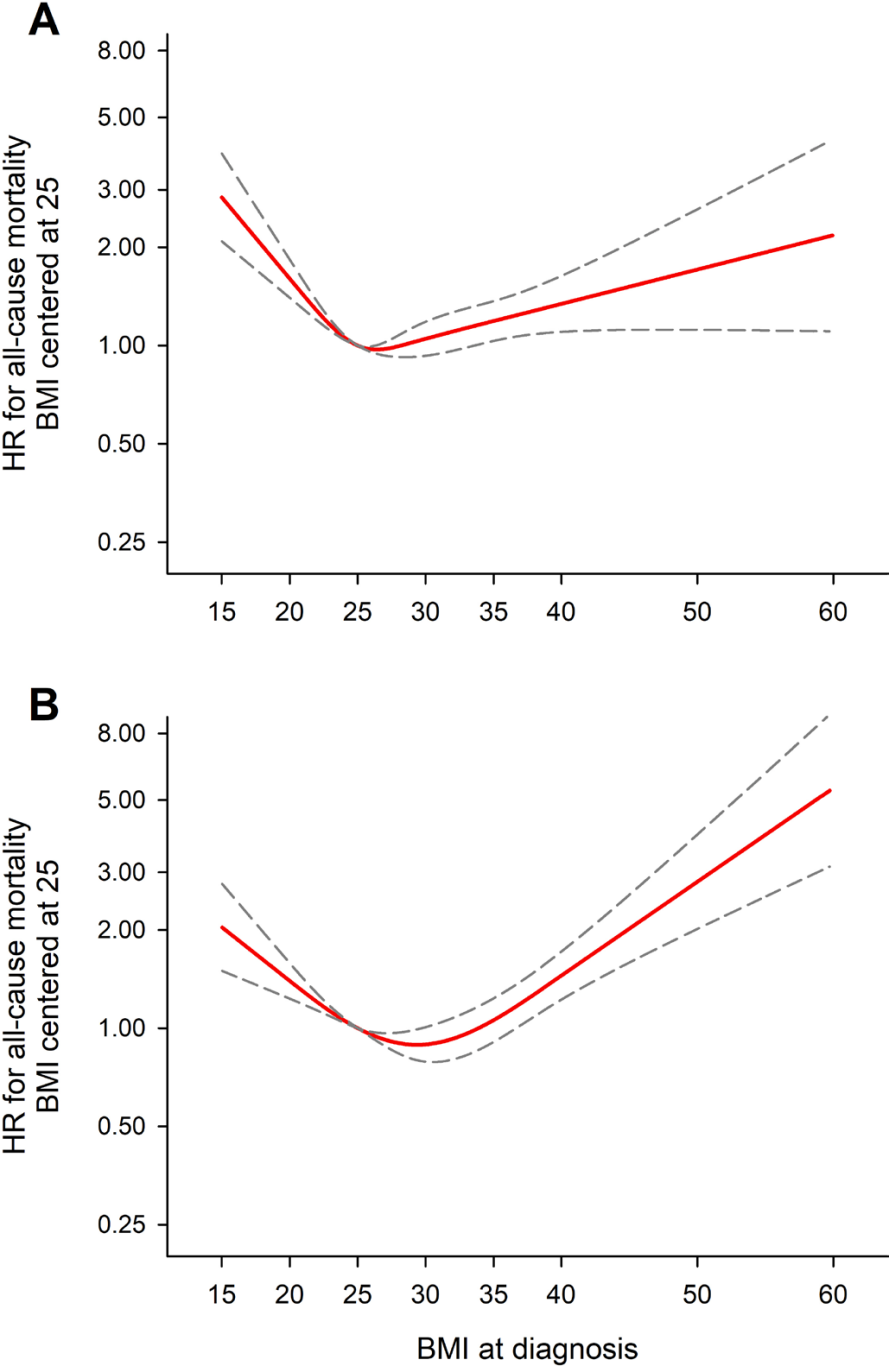
Adjusted^1^ associations through 2-year follow-up between BMI and selected endpoints based on a restricted cubic spline model in (A) male and (B) female patients.^1^ Adjusted by age, ethnicity, smoking status, alcohol use and moderate to severe CKD. BMI, body mass index; CKD, chronic kidney disease.

BMI showed similar association in both men and women with all-cause mortality; however, there were apparent differences in the strengths of association, p value for interaction assuming a linear trend: 0.01 (figure 5). While higher BMI appeared numerically more strongly associated with all-cause mortality in women (20%, 95% CI 11% to 30% increase per 5 kg/m^2^ higher BMI above 30 kg/m^2^) compared with men (11%, 95% CI 0% to 22% increase per 5 kg/m^2^ higher BMI above 30 kg/m^2^), the difference in this specific BMI range was not significant (p-interaction 0.32). Conversely, lower BMI (<30 kg/m^2^) was similarly associated with risk of all-cause mortality in women (15%, 95% CI 7% to 24% increase per 5 kg/m^2^ lower BMI below 30 kg/m^2^) and men (16%, 95% CI 8% to 25% decrease per 5 kg/m^2^ lower BMI below 30 kg/m^2^ (p-interaction 0.88).

### Sensitivity analysis

In patients without baseline heart failure, the association between BMI and weight and risk of new heart failure (330 events) was similar (online supplemental figure S3). No association was observed between height and risk of new heart failure. Age did not impact the association between BMI and risk of all-cause mortality or new/worsening heart failure with similar risk of outcomes observed in both younger and older participants (online supplemental figure S4).

Limiting the analysis to participants with at ≥6 months of follow-up (n=39 081) did not affect the association between BMI and AF outcomes (online supplemental figure S5). Furthermore, adjusting for baseline treatment did not alter these results (online supplemental figure S6).

**Figure 6:**
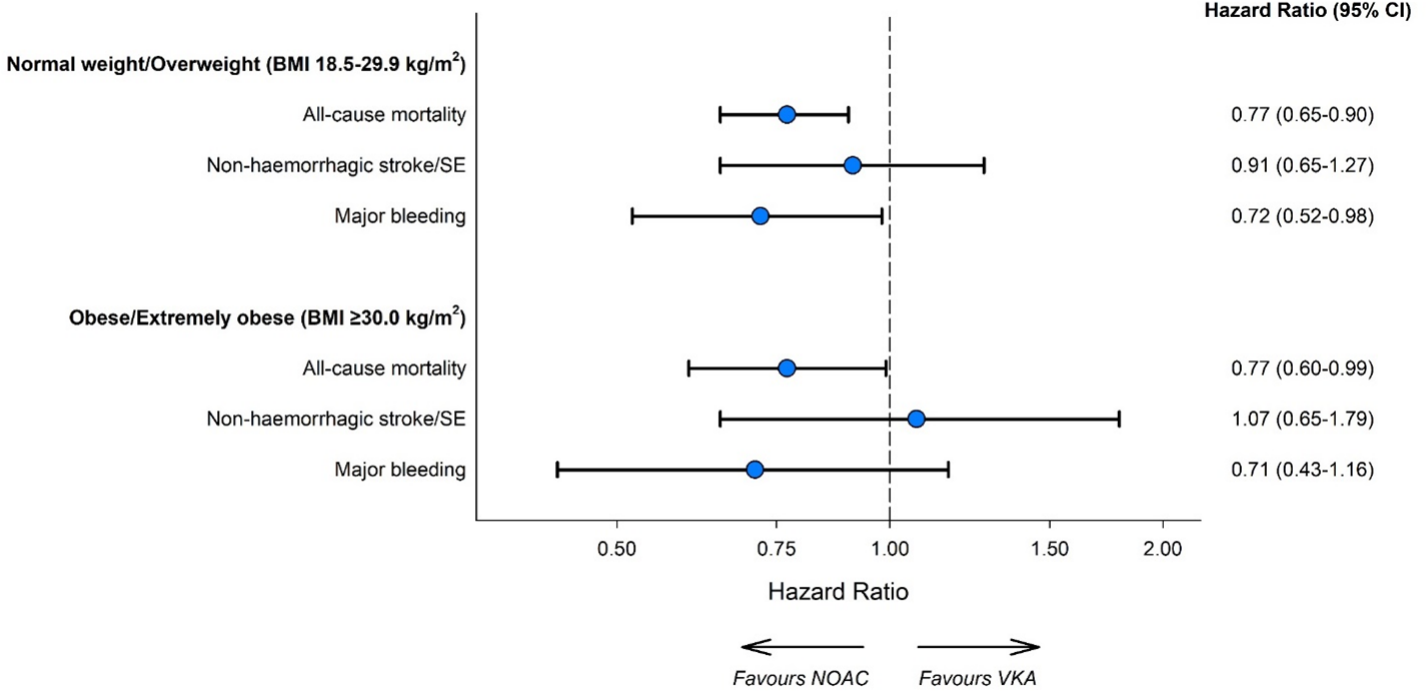
Propensity score weighted HRs* for NOAC versus VKA treatment through 2-year follow-up by BMI group. The reference category is patients treated with VKA treatment. *Obtained using an overlap-weighted Cox model. Variables included in the weighting scheme are: country and cohort enrolment, sex, age, ethnicity, type of AF, care setting specialty and location, congestive heart failure, acute coronary syndromes, vascular disease, carotid occlusive disease, prior stroke/TIA/SE, prior bleeding, VTE, hypertension, hypercholesterolaemia, diabetes, cirrhosis, moderate to severe CKD, dementia, hyperthyroidism, hypothyroidism, current smoking, heavy alcohol consumption, heart rate, systolic and diastolic blood pressure at diagnosis and baseline antiplatelet use. AF, atrial fibrillation; CKD, chronic kidney disease; NOAC, non-vitamin K oral anticoagulants; SE, systemic embolism; TIA; transient ischaemic attack; VKA, vitamin K antagonist.

Additional adjustment for a broader range of covariates (model 1: age, sex, ethnicity, smoking status, alcohol use and moderate to severe CKD; model 2: as model 1, plus hypertension, heart failure, diabetes, vascular disease, prior stroke/TIA/SE, history of bleeding and baseline anticoagulation) included suspected mediators of the association between higher BMI and risk of AF outcomes. This led to a substantial attenuation of the association between higher BMI and risk of all-cause mortality (4%, 95% CI −2 to 12% increase per 5 kg/m^2^ increase). Conversely, there was no meaningful difference in the negative association between low BMI (<30 kg/m^2^) and all-cause mortality (39%, 95% CI 31% to 47% increase per 5 kg/m^2^ decrease, online supplemental table S2).

### Effects of BMI in relation to NOAC therapy

Participants were separated into two groups: patients with a normal or overweight BMI (BMI 18.5–29.9 kg/m^2^, VKA participants=5051, NOAC participants=5612) and those with an obese/extremely obese BMI (BMI ≥30.0 kg/m^2^, VKA participants=2549, NOAC participants=2298). No significant difference was observed in the effect of NOAC therapy between BMI groups (figure 6). NOAC therapy was associated with lower all-cause mortality in both the normal/overweight group (HR 0.77, 95% CI 0.65 to 0.90) and the obese/extremely obese group (HR 0.77, 95% CI 0.60 to 0.99). There were no substantial differences in the impact of NOAC therapy on non-haemorrhagic stroke or major bleeding event risk between BMI groups.

## Discussion

This study demonstrated a non-linear association between BMI and weight, with risk of all-cause mortality and new/worsening heart failure. This suggested that those with both low and high values were at higher risk of these AF outcomes. There was no association of obesity/extreme obesity with the efficacy of NOACs versus VKAs when compared with a normal/overweight group.

Previously, an inverse relationship has been demonstrated between BMI and mortality in AF. Post hoc analysis of 21 028 ENGAGE TIMI-48 trial participants showed a ~10% lower risk of death and stroke per 5 kg/m^2^ higher BMI.^3^ Conversely a 6% higher risk of bleeding was demonstrated per 5 kg/m^2^ higher BMI. Similar findings have been reported from post hoc analysis of AFFIRM (n=2492) and ORBIT-AF participants (n=9513).^4 17^ Interestingly, results from GARFIELD-VTE similarly supported an ‘obesity paradox’ with all-cause mortality was lowest in obese patients.^18^ In contrast, Overvad *et al* 6 analysed a subgroup of the Danish Diet, Cancer, and Health cohort and, similar to our results, demonstrated a positive association with higher BMI and, intriguingly, a U-shaped relationship, which was particularly pronounced in women. Our findings support an inverse relationship between BMI and risk of death in AF but only for BMI <~30 kg/m^2^. Above this level, a strong positive association existed. While not in keeping with most studies examining AF patients, it is consistent with the association between BMI and mortality in the general population.^19^

The association of BMI with the risk of heart failure in AF has not been well established. Schnabel *et al* demonstrated a 6% higher risk of heart failure per 1 kg/m^2^ higher BMI in those with AF (AF cases=725).^20^ Assessment of the ORBIT-AF cohort, however, revealed no significant association between BMI and risk of heart failure.^17^ Our results demonstrated that both low and high BMI are associated with risk of new/worsening heart failure. Given the potential for reverse causation, we confirmed this when patients with events in the first 6 months of follow-up were excluded and in those without baseline heart failure.

Reasoning for the difference in our findings compared with prior groups is unclear. This study was limited to new-onset AF. Inclusion of participants previously diagnosed with AF, such as in ORBIT-AF and AFFIRM,^4 17^ may have led to a selection bias with those dying shortly after diagnosis being excluded. Additionally, previous studies have often adjusted for variables which may mediate relationships between BMI and mortality in patients with AF, such as hypertension/blood pressure, diabetes or history of ischaemic heart disease. Adjustment for variables involved in mediating pathways will necessarily bias results.^21^ Additional adjustments made in our analysis mitigated the positive association with higher BMI (>30 kg/m^2^), suggesting that over adjustment may have produced the previously reported negative association between BMI and mortality.

The mechanisms underlying the association between BMI and AF outcomes are not clearly established. Greater BMI has been associated with higher risk of hypertension,^22^ diabetes^23^ and coronary heart disease,^24^ which are established risk factors for mortality and heart failure. However, these comorbidities are associated with risk of ischaemic stroke,^25^ which was not associated with BMI in our analysis. As such, the mediating pathway between BMI and mortality/heart failure in AF may partially lie outside these established risk factors. Increasing adiposity has been associated with higher levels of epicardial fat and atrial fibrosis both of which are risk factors for AF.^26^ Further exploration into the role of cardiac factors in the association between BMI and AF outcomes may therefore be warranted.

Trend differences were observed in the BMI association with outcomes between sexes; high BMI was more strongly associated with mortality among women. However, the observed differences were numerically small, and more research is required to identify significant effect modifications between sexes.

Despite a strong association between height and risk of AF, there has been limited assessment of the association of height with AF-related outcomes. Height has previously been shown to be inversely associated with risk of stroke and cardiovascular disease.^27 28^ However, the association of height with cardioembolic stroke is not clear. A small case–control study in South Korea suggested a possible inverse association between height and stroke in those with AF.^29^ However, our findings have suggested no association between height and AF-related outcomes. Height is often considered a surrogate marker for lean mass. As such, these results may suggest that fat mass, rather than lean mass, is associated with higher mortality and heart failure in those with AF. This would contrast with AF risk itself where both lean and fat mass are associated.^2^ Our findings suggest a need for assessment of the association of lean and fat mass with risk of AF outcomes.

The causal relevance of this association between BMI and AF outcomes is not clear. However, previous trials of weight loss in patients with AF have demonstrated substantial improvements in symptoms and AF burden.^30^ To date, no randomised trial of weight loss in AF has been powered to detect meaningful changes in AF outcomes. Our results suggest that further assessment of weight loss in overweight/obese individuals with AF should be considered to determine the impact on reduced mortality and heart failure.

The potential detrimental effects of raised BMI on the efficacy of NOAC therapy have been raised previously.^11^ Robust pharmacokinetic and pharmacodynamics data in obesity and extreme obesity are lacking. Piran *et al* 31 suggested that one-fifth of extremely obese patients have a peak NOAC plasma concentration below the expected range. A meta-analysis of randomised controlled trials also suggested a similar rate of stroke and major bleeding in obese patients treated NOACs and warfarin.^32^ In line with this, our findings suggested there is no difference between NOAC and VKA therapy on the risk of non-haemorrhagic stroke or major bleeding in obese/extremely obese participants. In contrast, our results demonstrated substantially lower all-cause mortality with NOAC compared with VKA therapy in both normal/overweight and obese/extremely obese participants. This suggests that while there may be differences in the pharmacokinetics/pharmacodynamics of NOACs in obese/extremely obese individuals, NOAC therapy is likely more beneficial than VKA therapy in those with higher BMI.

This study has several strengths, including its large size, data on a broad range of major potential confounders and excellent participant follow-up. However, observational associations can be subject to uncontrolled residual confounding. Additionally, the lack of more detailed anthropometric measures limits our ability to make mechanistic inferences. As AF development is casually linked to BMI and a series of clinical endpoints, by restricting enrolment to AF patients, the obtained associations between BMI and clinical endpoints are possibly subject to collider stratification bias. Studies that collect longitudinal BMI measurements preceding AF incidence and the application of time-varying exposure methods would help quantify the magnitude of this bias.

## Conclusions

Our findings from this large-scale observational cohort suggest important associations between both high and low BMI and risk of mortality and new/worsening heart failure in those with AF. These findings are not in line with the so-called ‘obesity paradox’ and suggest that further investigation of weight management strategies in those with AF may reduce mortality and heart failure risk.

## Data Availability

All data relevant to the study are included in the article or uploaded as supplementary information.
