# Supplementary material for: Association of body mass index with outcomes in patients with newly diagnosed atrial fibrillation: GARFIELD-AF
Source: Open Heart. 2022 Aug 5;9(2):e002038. doi: 10.1136/openhrt-2022-002038 (PMC9362832; doi:10.1136/openhrt-2022-002038)
Supplement: Supplementary data [file openhrt-2022-002038supp001.pdf]

The association of body mass index with outcomes in patients with newly diagnosed atrial fibrillation – GARFIELD-AF

C. Fielder Camm[1], BM BCh, Saverio Virdone[2], MSc, Shinya Goto[3], MD, Jean-Pierre Bassand[2,4], MD, Martin van Eickels[5], MD, Sylvia Haas[6], MD, PhD, Bernard J. Gersh[7], MB, Karen S. Pieper[2], PhD, Keith A.A. Fox[8], MBChB, Frank Misselwitz[9], MD, PhD, Alexander G.G. Turpie[10], MD, Samuel Z. Goldhaber[11], MD, Freek W.A. Verheugt[12], MD, PhD, A. John Camm[13], MD, Gloria Kayani[2], BSc, Elizaveta Panchenko[14], PhD, Seil Oh[15], MD, Hector Lucas Luciardi[16], MD, Jitendra Pal Singh Sawhney[17], MD, Stuart Connolly[18], MD, Pantep Angchaisuksiri[19], MD, Hugo ten Cate[20], MD, PhD, John Eikelboom[21], MBBS, Ajay K. Kakkar, PhD[2,22] on behalf of the GARFIELD-AF investigators*

Camm CF, *et al. Open Heart* 2022; 9:e002038. doi: 10.1136/openhrt-2022-002038

BMJ Publishing Group Limited (BMJ) disclaims all liability and responsibility arising from any reliance placed on this supplemental material which has been supplied by the author(s)

Supplemental Material

Supplementary Table S1. Event rates (per 100 person-years) through 2-years of follow-up by BMI category.

Outcome	Events	Event Rate (95% CI)
**All-cause mortality**		
Underweight	117	9.62 (8.03-11.53)
Normal weight	1052	4.38 (4.13-4.66)
Overweight	927	3.28 (3.07-3.49)
Obese	460	3.21 (2.93-3.52)
Extremely Obese	249	3.22 (2.84-3.65)
**Non-haemorrhagic stroke/SE**		
Underweight	12	0.99 (0.56-1.75)
Normal weight	267	1.12 (1.00-1.27)
Overweight	295	1.05 (0.94-1.18)
Obese	130	0.91 (0.77-1.09)
Extremely Obese	66	0.86 (0.68-1.09)
**Major bleeding**		
Underweight	23	1.92 (1.28-2.89)
Normal weight	241	1.01 (0.89-1.15)
Overweight	258	0.92 (0.81-1.04)
Obese	137	0.97 (0.82-1.14)
Extremely Obese	71	0.93 (0.73-1.17)
**New/worsening heart failure**		
Underweight	13	1.08 (0.63-1.86)
Normal weight	142	0.60 (0.51-0.70)
Overweight	193	0.69 (0.60-0.79)
Obese	143	1.01 (0.86-1.19)
Extremely Obese	98	1.29 (1.06-1.57)

Camm CF, *et al. Open Heart* 2022; 9:e002038. doi: 10.1136/openhrt-2022-002038

Supplementary Table S2. BMI effects (HR and 95% CI per 5kg/m^2 increase) on selected outcomes using Cox models with different sets of adjustments.

**Outcomes**	**Model 1[1]**		**Model 2[2]**	
All-cause mortality	**<30 kg/m^2**	**≥30 kg/m^2**	**<30 kg/m^2**	**≥30 kg/m^2**
	1.32 (1.25-1.40)	1.16 (1.09-1.23)	1.39 (1.31-1.47)	1.04 (0.98-1.12)
Non-haemorrhagic stroke/SE	**Linear**		**Linear**	
	1.01 (0.94-1.07)		1.00 (0.93-1.07)	
Major bleeding	**<30 kg/m^2**	**≥30 kg/m^2**	**<30 kg/m^2**	**≥30 kg/m^2**
	1.12 (1.01-1.26)	1.10 (0.97-1.24)	1.17 (1.04-1.31)	1.07 (0.95-1.22)
New/worsening HR	**<25 kg/m^2**	**≥25 kg/m^2**	**<25 kg/m^2**	**≥25 kg/m^2**
	1.12 (0.96-1.32)	1.23 (1.14-1.33)	1.15 (0.99-1.36)	1.18 (1.09-1.29)

[1]Adjustments: age, sex, ethnicity, smoking status, alcohol use, and moderate to severe CKD;

[2]Adjustments: age, sex, ethnicity, smoking status, alcohol use, moderate to severe CKD, hypertension, heart failure, diabetes, vascular disease, prior stroke/TIA/SE, history of bleeding, baseline anticoagulation

Camm CF, *et al. Open Heart* 2022; 9:e002038. doi: 10.1136/openhrt-2022-002038

Supplementary Figure S1a. Absolute standardised differences for NOAC vs VKA comparison before and after propensity score weighting among patients with normal weight or overweight (BMI 18.5-29.9 kg/m^2)

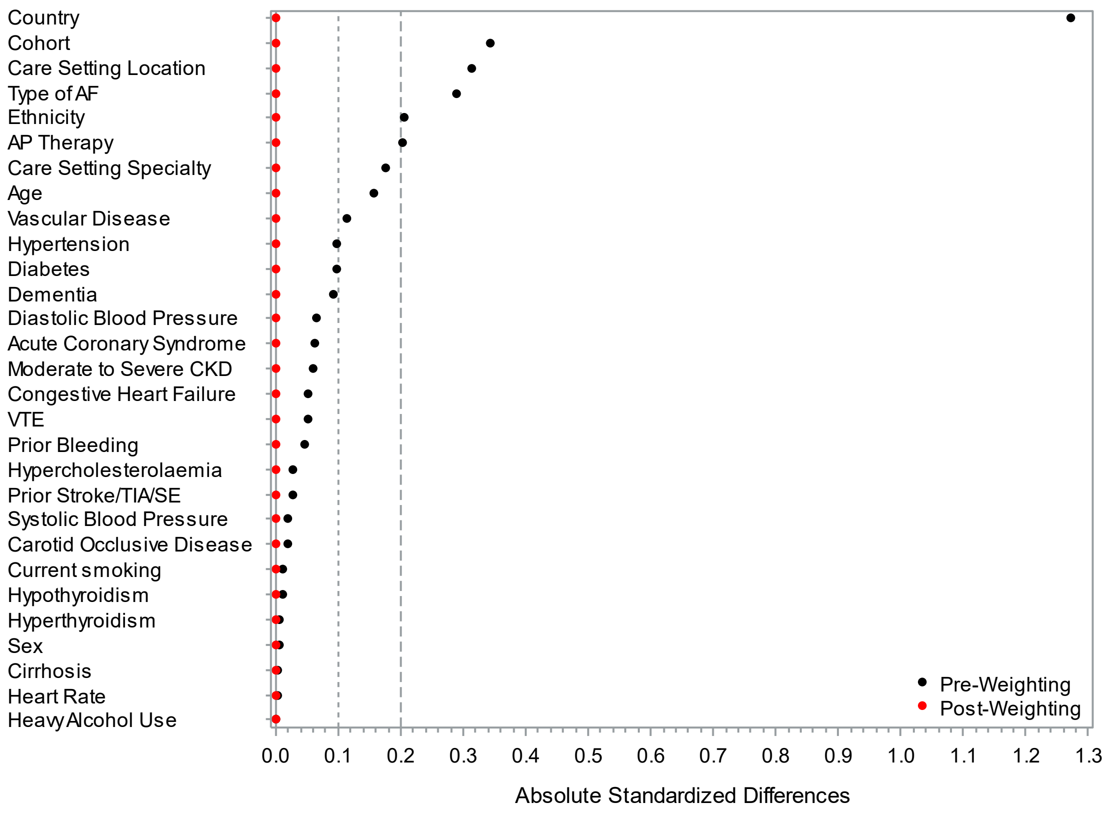

Supplementary Figure S1b. Absolute standardised differences for NOAC vs VKA comparison before and after propensity score weighting among patients obese or extremely obese (BMI ≥30 kg/m^2)

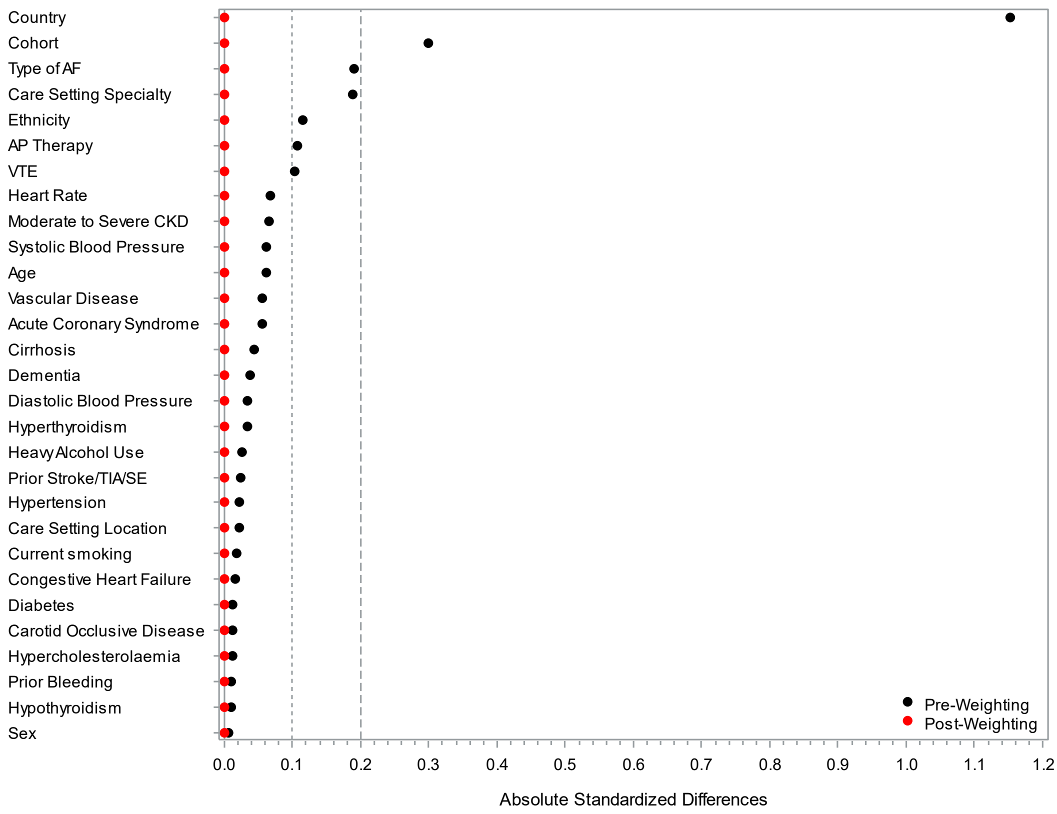

Supplementary Figure S2. Baseline treatment distribution[1] by BMI category among patients with CHA_2DS_2VASc score of ≥2.

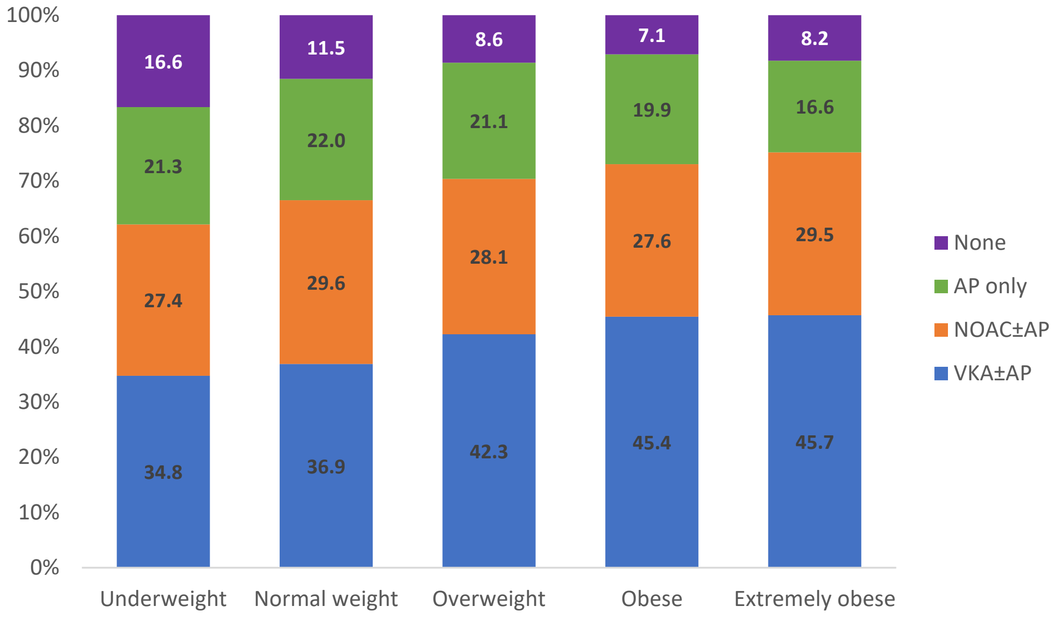

[1]A total of 518 patients were excluded from this analysis because of missing baseline treatment information

Camm CF, *et al. Open Heart* 2022; 9:e002038. doi: 10.1136/openhrt-2022-002038

BMJ Publishing Group Limited (BMJ) disclaims all liability and responsibility arising from any reliance placed on this supplemental material which has been supplied by the author(s)

Supplementary Figure S3. Adjusted[1] associations through 2-years follow-up between BMI and new heart failure based on a restricted cubic spline model.

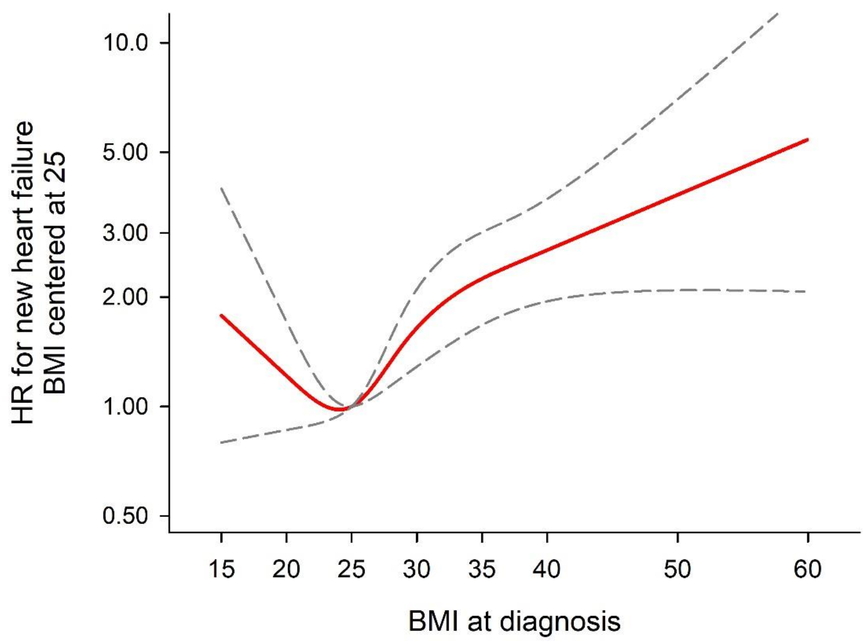

[1]Adjusted by age, sex, ethnicity, smoking status, alcohol use, and moderate to severe CKD.

Camm CF, *et al. Open Heart* 2022; 9:e002038. doi: 10.1136/openhrt-2022-002038

Supplementary Figure S4. Adjusted[1] hazard ratios through 2-years follow-up by BMI category for ages 65 and 80. The reference category is normal weight

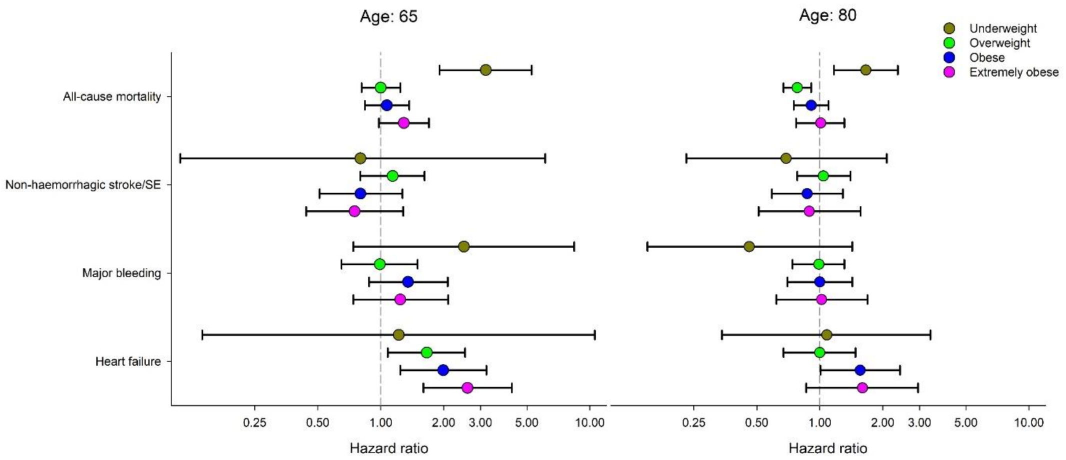

[1]Adjusted by age, sex, ethnicity, smoking status, alcohol use, and moderate to severe CKD. Age included using restricted cubic spline with 4 knots.

Supplementary Figure S5. Adjusted[1] associations through 2-years follow-up between BMI and selected endpoints based on a restricted cubic spline model in patients with at least 6 months of follow-up (n=39,081).

[1]Adjusted by age, sex, ethnicity, smoking status, alcohol use, and moderate to severe CKD.

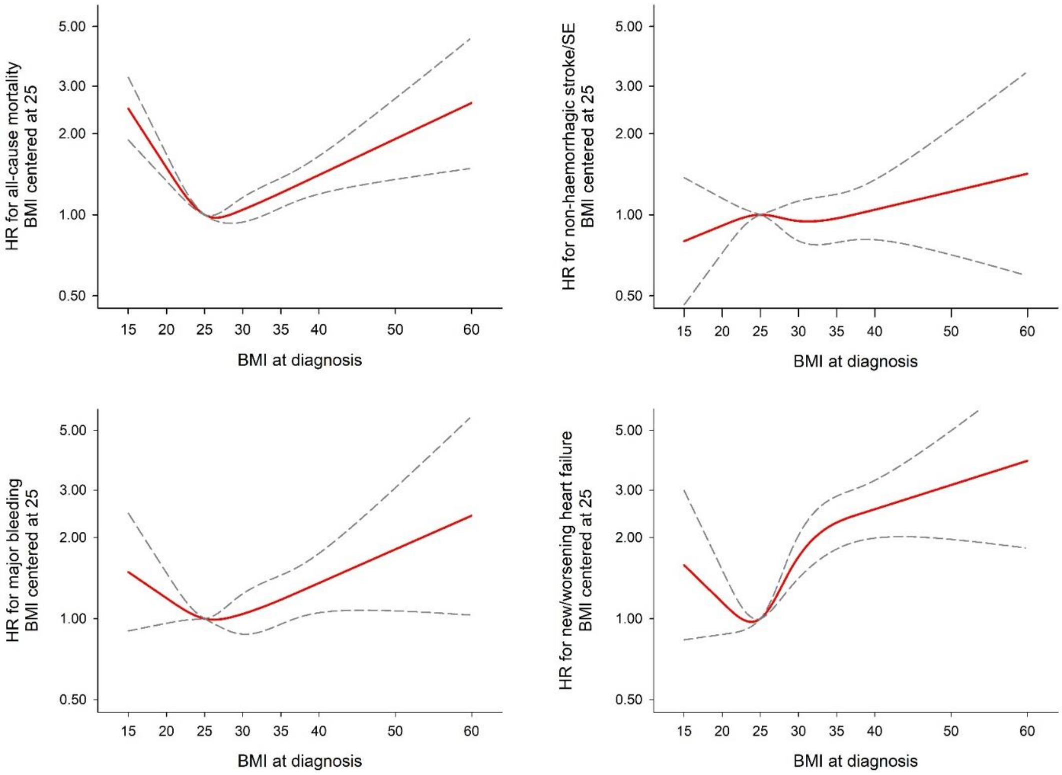

Supplementary Figure S6. Adjusted[1] associations through 2-years follow-up between BMI and selected endpoints based on a restricted cubic spline model

[1]Adjusted by age, sex, ethnicity, baseline treatment, smoking status, alcohol use, and moderate-to-severe CKD.

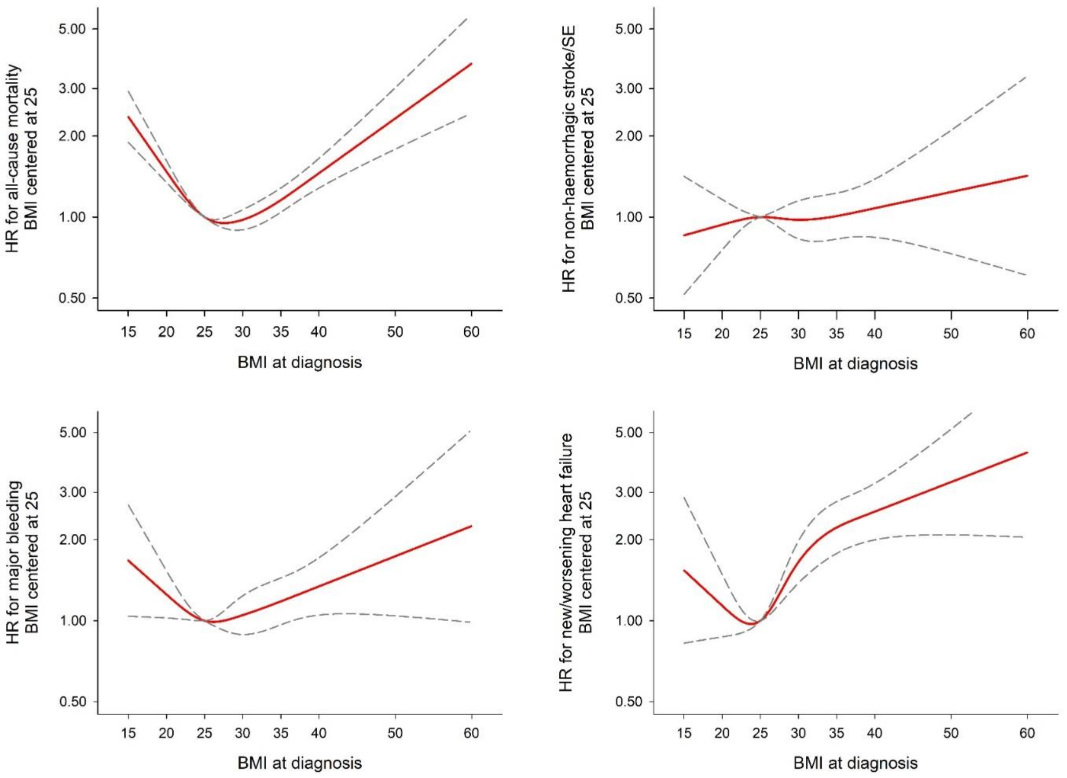

GARFIELD-AF Registry Investigators

Global Steering Committee

Ajay K. Kakkar (UK) (Chair), Jean-Pierre Bassand (France), A. John Camm (UK), David A. Fitzmaurice (UK), Keith A.A. Fox (UK), Bernard J. Gersh (USA), Samuel Z. Goldhaber (USA), Shinya Goto (Japan), Sylvia Haas (Germany), Werner Hacke (Germany), Lorenzo G. Mantovani (Italy), Frank Misselwitz (Germany), Karen S. Pieper (USA), Alexander G.G. Turpie (Canada), Martin van Eickels (Germany), Freek W.A. Verheugt (the Netherlands).

Audit Committee

Keith A.A. Fox (UK), Bernard J. Gersh (USA).

GARFIELD-AF National Coordinators:

Hector Lucas Luciardi (Argentina), Harry Gibbs (Australia), Marianne Brodmann (Austria), Frank Cools (Belgium), Antonio Carlos Pereira Barretto (Brazil), Stuart J. Connolly, John Eikelboom (Canada), Ramon Corbalan (Chile), Zhi-Cheng Jing (China), Petr Jansky (Czech Republic), Jørn Dalsgaard Nielsen (Denmark), Hany Ragy (Egypt), Pekka Raatikainen (Finland), Jean-Yves Le Heuzey (France), Harald Darius (Germany), Matyas Keltai (Hungary), Jitendra Pal Singh Sawhney (India), Giancarlo Agnelli and Giuseppe Ambrosio (Italy), Yukihiro Koretsune (Japan), Carlos Jerjes Sánchez Díaz (Mexico), Hugo Ten Cate (the Netherlands), Dan Atar (Norway), Janina Stepinska (Poland), Elizaveta Panchenko (Russia), Toon Wei Lim (Singapore), Barry Jacobson (South Africa), Seil Oh (South Korea), Xavier Viñolas (Spain), Marten Rosenqvist (Sweden), Jan Steffel (Switzerland), Pantep Angchaisuksiri (Thailand), Ali Oto (Turkey), Alex Parkhomenko (Ukraine), Wael Al Mahmeed (United Arab Emirates), David Fitzmaurice (UK), Samuel Z. Goldhaber (USA).

GARFIELD-AF National Investigators:

China

Dayi Hu, Kangning Chen, Yusheng Zhao, Huaiqin Zhang, Jiyan Chen, Shiping Cao, Daowen Wang, Yuejin Yang, Weihua Li, Hui Li, Yuehui Yin, Guizhou Tao, Ping Yang, Yingmin Chen, Shenghu He, Yong Wang, Guosheng Fu, Xin Li, Tongguo Wu, Xiaoshu Cheng, Xiaowei Yan, Ruiping Zhao, Moshui Chen, Longgen Xiong, Ping Chen, Yang Jiao, Ying Guo, Li Xue, Zhiming Yang.

India

Praveen Jadhavm, Raghava Sarma, Govind Kulkarni, Prakash Chandwani, Rasesh Atulbhai Pothiwala, Mohanan Padinhare Purayil, Kamaldeep Chawla, Veerappa Annasaheb Kothiwale, Bagirath Raghuraman, Vinod Madan Vijan, Jitendra Sawhney, Ganapathi Bantwal, Aziz Khan, Ramdhan Meena, Manojkumar Chopada, Sunitha Abraham, Vikas Bisne, Govindan Vijayaraghavan, Debabrata Roy, Rajashekhar Durgaprasad, A.G. Ravi Shankar, Sunil Kumar, Dinesh Jain, Kartikeya Bhargava, Vinay Kumar, Udigala Madappa Nagamalesh, Rajeeve Kumar Rajput.

Japan

Yukihiro Koretsune, Seishu Kanamori, Kenichi Yamamoto, Koichiro Kumagai, Yosuke Katsuda, Keiki Yoshida, Fumitoshi Toyota, Yuji Mizuno, Ikuo Misumi, Hiroo Noguchi, Shinichi Ando, Tetsuro Suetsugu, Masahiro Minamoto, Hiroyuki Oda, Susumu Adachi, Kei Chiba, Hiroaki Norita, Makoto Tsuruta, Takeshi Koyanagi, Kunihiko Yamamoto, Hiroshi Ando, Takayuki Higashi, Megumi Okada, Shiro Azakami, Shinichiro Komaki, Kenshi Kumeda, Takashi Murayama, Jun Matsumura, Yurika Oba, Ryuji Sonoda, Kazuo Goto, Kotaro Minoda, Yoshikuni Haraguchi, Hisakazu Suefuji, Hiroo Miyagi, Hitoshi Kato, Tsugihiro Nakamura, Tadashi Nakamura, Hidekazu Nandate, Ryuji Zaitsu, Yoshihisa Fujiura, Akira Yoshimura, Hiroyuki Numata, Jun Ogawa, Yasuyuki Kamogawa, Kinshiro Murakami, Yutaka Wakasa, Masanori Yamasawa, Hiromitsu Maekawa, Sumihisa Abe, Hajime Kihara, Satoru Tsunoda, Katsumi Saito, Hiroki Tachibana, Ichiro Oba, Takashi Kuwahata, Satoshi Higa, Masamichi Gushiken, Takuma Eto, Hidetoshi Chibana, Kazuaki Fujisawa, Yuhei Shiga, Hirokuni Sumi, Toshihisa Nagatomo, Yoshihiko Atsuchi, Toshiro Nagoshi, Kazuhisa Sanno, Fumihiro Hoshino, Naoto Yokota, Masahiro Kameko, Toshifumi Tabuchi, Munesumi Ishizawa, Yoshitake Fujiura, Daisuke Ikeda,Taku Seto, Tetsu Iwao, Norio Ishioka, Koichi Oshiro, Keizo Tsuchida, Yutaka Hatori, Motoshi Takeuchi, Hiroto Takezawa, Shinjiro Nagano, Masaaki Iwaki, Yuichiro Nakamura, Naomasa Miyamoto, Toshifumi Taguchi, Ko Ashida, Naoto Yoshizawa, Jun Agata, Seishiro Matsukawa, Osamu Arasaki, Shuji Fukuoka, Hirofumi Murakami, Kazuya Mishima, Yoshiki Hata, Ichiro Sakuma, Kotaro Obunai, Ichiro Takamura, Mitsuyuki Akutsu, Toshihide Unoki, Yoshinori Go, Makoto Ikemura, Shoji Morii, Shigeru Marusaki, Hideo Doi, Mitsuru Tanaka, Takaaki Kusumoto, Shigeo Kakinoki, Chiga Ogurusu, Kazuya Murata, Masaki Shimoyama, Masami Nakatsuka, Yutaka Kitami, Yoichi Nakamura, Hiroshi Oda, Rikimaru Oyama, Masato Ageta, Teruaki Mita, Kazuhiko Nagao, Takafumi Mito, Junichi Minami, Mitsunori Abe, Masako Fujii, Makoto Okawa, Tsuneo Fujito, Toshiya Taniguchi, Tenei Ko,

Hiroshi Kubo, Mizuho Imamaki, Masahiro Akiyama, Takashi Ueda, Hironori Odakura, Masahiko Inagaki, Yoshiki Katsube, Atsuyuki Nakata, Shinobu Tomimoto, Mitsuhiro Shibuya, Masayuki Nakano, Kenichiro Ito, Masahiro Matsuta, Motoyuki Ishiguro, Taro Minagawa, Masamichi Wada, Hiroaki Mukawa, Masato Mizuguchi, Fumio Okuda, Teruaki Kimura, Kuniaki Taga, Masaaki Techigawara, Morio Igarashi, Hiroshi Watanabe, Toshihiko Seo, Shinya Hiramitsu, Hiroaki Hosokawa, Mitsumoto Hoshiai, Michitaka Hibino, Koichi Miyagawa, Hideki Horie, Nobuyoshi Sugishita, Yukio Shiga, Akira Soma, Kazuo Neya, Tetsuro Yoshida, Kunio Akahane, Sen Adachi, Chiei Takanaka, Takashi Ueda, Saori Matsui, Hirofumi Kanda, Masanori Kaneko, Shiro Nagasaka, Atsushi Taguchi, Shuta Toru, Kazuyuki Saito, Akiko Miyashita, Hiroki Sasaguri, Jin Nariyama, Taketo Hatsuno, Takash Iwase, Kazuki Sato, Kazuya Kawai, Tomobumi Kotani, Tsuyoshi Tsuji, Hirosumi Sakai, Kiyoshi Nishino, Kenichi Ikeda, Kazuo Maeda, Tomohiro Shinozuka, Takeshi Inoue, Koichi Kawakami, Hiromichi Kitazumi, Tsutomu Takagi, Mamoru Hamaoka, Jisho Kojima, Akitoshi Sasaki, Yoshihiro Tsuchiya, Tetsuo Betsuyaku, Koji Higuchi, Masaaki Honda, Koichi Hasegawa, Takao Baba, Kazuaki Mineoi, Toshihiko Koeda, Kunihiko Hirasawa, Toshihide Kumazaki, Akira Nakagomi, Eiji Otaki, Takashi Shindo, Hiroyoshi Hirayama, Chikako Sugimoto, Takashi Yamagishi, Ichiro Mizuguchi, Kazunori Sezaki, Isamu Niwa, Ken Takenaka, Osamu Iiji, Koichi Taya, Hitoshi Kitazawa, samu Ueda, Hirokazu Kakuda, Takuya Ono, Seizo Oriso, Junya Kamata, Toshihiko Nanke, Itaru Maeda, Yoshifusa Matsuura, Hiroki Teragawa, Yasuyuki Maruyama, Kazuo Takei, Hajime Horie, Tetsutaro Kito, Hiroshi Asano, Koji Matsushita, Masaichi Nakamura, Takashi Washizuka, Tomoki Yoshida, Masato Sawano, Shinichi Arima, Hidekazu Arai, Hisanori Shinohara, Hiroyuki Takai, Nobufusa Furukawa, Akira Ota, Kentaro Yamamoto, Kenji Aoki, Taku Yamamoto, Takeaki Kasai, Shunji Suzuki, Shu Suzuki, Nitaro Shibata, Masayuki Watanabe, Yosuke Nishihata, Toru Arino, Masaki Okuyama, Tetsushi Wakiyama, Tomoko Kato, Yasuo Sasagawa, Takeshi Tana, Yoshihito Hayashi, Shinichi Hirota, Yukihiko Abe, Yoshihiro Saito, Hirohide Uchiyama, Hiroshi Takeda, Hiroshi Ono, Shuichi Tohyo, Naoto Hanazono, Seiichi Miyajima, Hisashi Shimono, Takuma Aoyama, Yasunobu Shozawa, Yawara Niijima, Osamu Murai, Osamu Murai, Hideko Inaba, Katsumasa Nomura, Masatsugu Nozoe, Kazuo Suzuki, Toshiyuki Furukawa, Toshihiko Shiraiwa, Nobuhisa Ito, Shunichi Nagai, Kiyoharu Sato, Shiro Nakahara, Yujin Shimoyama, Naoko Ohara, Teruhiko Kozuka, Hideaki Okita, Masato Endo, Tsutomu Goto, Makoto Hirose, Emiko Nagata, Noriyuki Nakanishi, Toshizumi Mori, Shuichi Seki, Katsuhiro Okamoto, Osamu Moriai, Yoko Emura, Tsuyoshi Fukuda, Haruhiko Date, Shuichi Kawakami, Sho Nagai, Yuya Ueyama, Tetsuro Fudo, Mitsuru Imaizumi, Takuo Ogawa, Shunsuke Take, Hideo Ikeda, Hiroaki Nishioka, Norihiko Sakamoto, Kiyomitsu Ikeoka, Nobuo Wakaki, Masatake Abe, Junji Doiuchi, Tetsuya Kira, Masato Tada, Ken Tsuzaki, Naoya Miura, Yasuaki Fujisawa, Wataru Furumoto, Susumu Suzuki, Akinori

Fujisawa, Ryosai Nakamura, Hiroyasu Komatsu, Rei Fujiki, Shuichi Kawano, Keijiro Nishizawa, Yoji Kato, Junya Azuma, Kiyoshi Yasui,Toshio Amano, Yasuhiro Sekine, Tatsuo Honzawa, Yuichiro Koshibu, Yasuhide Sakamoto, Yukihiro Seta, Shingo Miyaguchi, Kojuro Morishita, Yasuko Samejima, Toyoshi Sasaki, Fumiko Iseki, Toshiyuki Kobayashi, Hiroshi Kano, Jaeyoung Kim, Hiroshi Yamaguchi, Yoichi Takagi, Yoko Onuki Pearce, Yasuyuki Suzuki, Takayuki Fukui, Toru Nakayama, Hideaki Kanai, Yoshiyuki Kawano, Tetsuji Ino, Hironori Miyoshi, Yasufumi Miyamoto, Masahito Shigekiyo, Shimato Ono, Yoshiyuki Kawano, Yutaka Okamoto, Satoshi Ubukata, Kojiro Kodera, Tatsuo Oriuchi, Naoki Matsumoto, Koichi Inagaki, Atsushi Iseki, Tomohiro Yoshida, Toshihiro Goda, Tsukasa Katsuki, Atsushi Sato, Etsuo Mori, Toshio Tsubokura, Hiroshi Shudo, Shunichi Fujimoto, Tomohiro Katsuya, Yoshiyuki Furukawa, Hiroshi Hosokawa, Jun Narumi, Kiichiro Yamamoto, Masaki Owari, Takuya Inakura, Takafumi Anno, Kazuyuki Shirakawa.

Singapore

Chi Keong Ching, Toon Wei Lim, David Foo, Kelvin Wong, Tan Yuyang.

South Korea

Seil Oh, Hui Nam Park, Woo-Shik Kim, HyeYoung Lee, Sung-Won Jang, Dae Hyeok Kim, Jun Kim, DongRyeol Ryu, Jaemin Shim, Dae-Kyeong Kim, Dong Ju Choi, Yong Seog Oh, Myeong-Chan Cho, Hack-Lyoung Kim, Hui-Kyung Jeon, Dong-Gu Shin, Sang Weon Park, Hoon Ki Park, Sang-Jin Han, Jung Hoon Sung, Hyung-Wook Park, Gi-Byoung Nam, Young Keun On, Hong Euy Lim, JaeJin Kwak, Tae-Joon Cha, Taek Jong Hong, Seong Hoon Park, Jung Han Yoon, Nam-Ho Kim, Kee-Sik Kim, Byung Chun Jung, Gyo-Seung Hwang, Chong-Jin Kim.

Thailand

Sakda Rungaramsin, Peerapat Katekangplu, Porames Khunrong, Thanita Bunyapipat, Wanwarang Wongcharoen, Pinij Kaewsuwanna, Khanchai Siriwattana, Waraporn Tiyanon, Supalerk Pattanaprichakul, Khanchit Likittanasombat, Doungrat Cholsaringkarl, Warangkana Boonyapisit, Sirichai Cheewatanakornkul, Songkwan Silaruks, Pisit Hutayanon, Seksan Chawanadelert, Pairoj Chattranukulchai, Boonsert Chatlaong, Yingsak Santanakorn, Khompiya Kanokphatcharakun, Piya Mongkolwongroj, Sasivimon Jai-Aue, Ongkarn Komson.

Turkey

Armagan Altun, Ali Aydinlar, Ramazan Topsakal, Zeki Ongen, Sadik Acikel, Durmus Yildiray Sahin, Ozcan Yilmaz, Mehmet Birhan Yilmaz, Hasan Pekdemir, Mesut Demir, Murat Sucu, Levent Sahiner, Ali Oto, Murat Ersanli, Ertugrul Okuyan, Dursun Aras.

Argentina

Florencia Rolandi, Adrian Cesar Ingaramo, Gustavo Alberto Sambadaro, Vanina Fernandez Caputi, Hector Luciardi, Sofia Graciela Berman, Pablo Dragotto, Andres Javier Kleiban, Nestor Centurion, Rodolfo Andres Ahuad Guerrero, Leonel Adalberto Di Paola, Ricardo Dario Dran, Javier Egido, Matias Jose Fosco, Victor Alfredo Sinisi, Luis Rodolfo Cartasegna, Oscar Gomez Vilamajo, Jose Luis Ramos, Sonia Sassone, Gerardo Zapata, Diego Conde, Guillermo Giacomi, Alberto Alfredo Fernandez, Mario Alberto Berli, Fabian Ferroni.

Brazil

Dário Celestino Sobral Filho, Jefferson Jaber, Luciana Vidal Armaganijan, Costantino Roberto Frack Costantini, André Steffens, Weimar Kunz Sebba Barroso de Souzaem, João David de Souza Neto, José Márcio Ribeiro, Marcelo Silveira Teixeira, Paulo Rossi, Leonardo Pires, Daniel Moreira, José Carlos Moura Jorge, Adalberto Menezes Lorga Filho, Luiz Bodanese, Marcelo Westerlund Montera, Carlos Henrique Del Carlo, Jamil Abdalla Saad, Fernando Augusto Alves da Costa, Renato Lopes, Gilson Roberto de Araújo, Euler Roberto Manenti, Jose Francisco Kerr Saraiva, João Carlos Ferreira Braga, Alexandre Negri, Carlos Moncada, Dalton Precoma, Fernando Roquette, Gilmar Reis, Roberto Álvaro Ramos Filho,: Estêvão Lanna Figueiredo, Roberto Vieira Botelho, Cláudio Munhoz da Fontoura Tavares, Helius Carlos Finimundi, Adriano Kochi, César Cássio Broilo França, Fábio Alban, Guido Bernardo Aranha Rosito, João Batista de Moura Xavier Moraes Junior, Rogério Tadeu Tumelero, Lilia Maia,: Roberto Simões de Almeida, Ney Carter do Carmo Borges, Luís Gustavo Gomes Ferreira.

Chile

Ramón Corbalán, Benjamin Aleck Joseh Stockins Fernandez, Humberto Montecinos, Fernando Lanas, Martín Larico Gómez, Carlos Astudillo, Carlos Conejeros, Patricio Marin Cuevas, Alejandro Forero, Claudio Bugueño Gutiérrez, Juan Aguilar, Sergio Potthoff Cardenas, German Eggers, Cesar Houzvic, Carlos Rey, Germán Arriagada, Gustavo Charme Vilches.

Mexico

Camm CF, *et al. Open Heart* 2022; 9:e002038. doi: 10.1136/openhrt-2022-002038

Carlos Jerjes Sanchez Diaz, Jesus Jaime Illescas Diaz, Raul Leal Cantu, Maria Guadalupe Ramos Zavala, Ricardo Cabrera Jardines, Nilda Espinola Zavaleta, Enrique Lopez Rosas, Guillermo Antonio Llamas Esperón, Gerardo Pozas, Ernesto Cardona Muñoz, Norberto Matadamas Hernandez, Adolfo Leyva Rendon, Norberto Garcia Hernandez, Manuel de los Rios Ibarra, Luis Ramon Virgen Carrillo,

David Lopez Villezca, Carlos Hernandez Herrera, Juan Jose Lopez Prieto, Rodolfo Gaona Rodriguez, Efrain Villeda Espinosa, David Flores Martinez, Jose Velasco Barcena, Omar Fierro Fierro, Ignacio Rodriguez Briones, Jose Luis Leiva Pons, Humberto Alvarez Lopez, Rafael Olvera Ruiz, Carlos Gerardo Cantu Brito, Eduardo Julian Jose Roberto Chuquiure Valenzuela, Roxana Reyes Sanchez, Alberto Esteban Bazzoni Ruiz, Oscar Martin Lopez Ruiz, Roberto Arriaga Nava, Jesus David Morales Cerda, Pedro Fajardo Campos, Mario Benavides Gonzalez.

Austria

Marianne Brodmann, Kurt Lenz, Claus Hagn, Johannes Foechterle, Heinz Drexel, Kurt Huber, Andrea Podczeck-Schweighofer, Michael Winkler, Bruno Schneeweiss, Alfons Gegenhuber, Wilfried Lang, Sabine Eichinger-Hasenauer, Peter Kaserer, Josef Sykora, Heribert Rasch, Bernhard Strohmer.

Belgium

Luc Capiau, Geert Vervoort, Bart Wollaert, Frank Cools, Geert Hollanders, Jan Vercammen, Dirk Faes, Yohan Balthazar, Marc Delforge, Olivier Xhaet, Harry Striekwold, John Thoeng, Kurt Hermans, Georges Mairesse, Wim Anné, Ivan Blankoff, Michel Beutels, Stefan Verstraete, Peter Vandergoten, Philippe Purnode, Pascal Godart, Tim Boussy, Philippe Desfontaines, Alex Heyse, Joeri Voet, Axel De Wolf.

Czech Republic

Eva Zidkova, Petr Jansky, Rudolf Spacek, Vilma Machova, Ondrej Ludka, Josef Olsr, Lubos Kotik, Blazej Racz, Richard Ferkl, Jan Hubac, Ilja Kotik, Zdenek Monhart, Hana Burianova, Ondrej Jerabek, Jana Pisova, Iveta Petrova, Vratislav Dedek, Michaela Honkova, Petr Podrazil, Petr Reichert, Jindrich Spinar, Miroslav Novak, Vaclav Durdil, Katarina Plocova, Jiri Lastuvka.

Denmark

Camm CF, *et al. Open Heart* 2022; 9:e002038. doi: 10.1136/openhrt-2022-002038

Jørn Nielsen, Steen Husted, Helena Dominguez, Ulrik Hintze, Søren Rasmussen, Næstved Sygehus, Arne Bremmelgaard, John Markenvard, Jan Børger, Jorgen Solgaard, Ebbe Eriksen, Thomas Løkkegaard, Michael Bruun, Jacob Mertz, Morten Schou, Helena Dominguez, Michael Olsen.

Finland

Pekka Raatikainen, Carmela Viitanen.

France

Franck Paganelli, Joël Ohayon, Frédéric Casassus, Jean-Yves Le Heuzey, Michel Galinier, Yannick Gottwalles, Philippe Loiselet, Jean-Joseph Muller, Mohamed Bassel Koujan, André Marquand, Sylvain Destrac, Olivier Piot, Nicolas Delarche, Jean-Pierre Cebron, Maxime Guenoun, Dominique Guedj-Meynier, Lokesh A G, Mathieu Zuber, Pierre Amarenco, Emmanuel Ellie, James Kadouch, Pierre-Yves Fournier, Jean-Pierre Huberman, Nestor Lemaire, Gilles Rodier, Xavier Vandamme, Igor Sibon, Jean-Philippe Neau, Marie Hélène Mahagne, Antoine Mielot, Marc Bonnefoy, Jean-Baptiste Churet, Vincent Navarre, Frederic Sellem, Gilles Monniot, Jean-Paul Boyes, Bernard Doucet, Michel Martelet, Désiré Obadia, Bernard Crousillat, Joseph Mouallem, Etienne Bearez, Jean Philippe Brugnaux, Alain Fedorowsky, Pierre Nazeyrollas, Jean-Baptiste Berneau, Frédéric Chemin.

Germany

Sebastien Schellong, Harald Darius, Georg Koeniger, Andreas Kopf, Uwe Gerbaulet, Bernd-Thomas Kellner, Thomas Schaefer, Jan Purr, Enno Eißfeller, Heinz-Dieter Zauzig, Peter Riegel, Christoph Axthelm, Gerd-Ulrich Heinz, Holger Menke, Andreas Pustelnik, Stefan Zutz, Wolfgang Eder, Guenter Rehling, Dirk Glatzel, Norbert Ludwig, Petra Sandow, Henning Wiswedel, Cosmas Wildenauer, Steffen Schoen, Toralf Schwarz, Adyeri Babyesiza, Maximilian Kropp, Hans-Hermann Zimny, Friedhelm Kahl, Andreas Caspar, Sabine Omankowsky, Torsten Laessig, Hermann-Josef Hartmann, Gunter Lehmann, Hans-Walter Bindig, Gunter Hergdt, Dietrich Reimer, Joachim Hauk, Holger Michel, Praxis Dres. Werner Erdle, Wilfried Dorsch, Janna Dshabrailov, Karl-Albrecht Rapp, Reinhold Vormann, Thomas Mueller, Peter Mayer, Uwe Horstmeier, Volker Eissing, Heinz Hey, Heinz Leuchtgens, Volker Lilienweiss, Heiner Mueller, Christian Schubert, Herrmann Lauer, Thomas Buchner, Gunter Brauer, Susanne Kamin, Karsten Mueller, Sylvia Baumbach, Muwafeg Abdel-Qader, Hans-Holger Ebert, Carsten Schwencke, Peter Bernhardt, Laszlo Karolyi, Britta Sievers, Wilhelm Haverkamp, Jens-Uwe Roehnisch.

Hungary

Andras Vertes, Gabor Szantai, Andras Matoltsy, Nikosz Kanakaridisz, Zoltan Boda, Erno Kis, Balazs Gaszner, Ferenc Juhasz, Gizella Juhasz, Sandor Kancz, Zoltan Laszlo, Zsolt May, Bela Merkely, Ebrahim Noori, Tamas Habon, Peter Polgar, Gabriella Szalai, Sandor Vangel, Andras Nagy, Gabriella Engelthaler, Judit Ferenczi, Mihaly Egyutt.

Italy

Giuliana Martini, Leone Maria Cristina, Eros Tiraferri, Rita Santoro, Sophie Testa, Giovanni Di Minno, Marco Moia, Teresa Maria Caimi, Maria Tessitori, Giancarlo Agnelli, Roberto Cappelli, Daniela Poli, Roberto Quintavalla, Franco Cosmi, Raffaele Fanelli, Vincenzo Oriana, Raffaele Reggio, Roberto Santi, Leonardo Pancaldi, Raimondo De Cristofaro, Giuliana Guazzaloca, Angelo De Blasio, Jorge Salerno Uriate, Flavia Lillo, Enrico Maria Pogliani, Grzegorz Bilo, Michele Accogli, Antonio Mariani, Mauro Feola, Arturo Raisaro, Luciano Fattore, Andrea Mauric, Fabrizio Germini, Luca Tedeschi, Maria Settimi, Sergio Nicoli, Paolo Ricciarini, Antonio Argena, Paolo Ronchini, Claudio Bulla, Filippo Tradati, Massimo Volpe, Maria D'Avino, Maria Grazia Bongiorni, Silva Severi, Alessandro Capucci, Corrado Lodigiani, Enrico Salomone, Gaetano Serviddio, Claudio Tondo, Giuseppe Ambrosio, Paolo Golino, Carmine Mazzone, Saverio Iacopino.

The Netherlands

Hugo ten Cate, J.H. Ruiter, Andreas Lucassen, Henk Adriaansen, Maarten Bongaerts, Mathijs Pieterse, Coen van Guldener, Johannes Herrman, S.H.K. P.R. Nierop, Pieter Hoogslag, Walter Hermans, B.E. Groenemeijer, W. Terpstra, Cees Buiks, L.V.A. Boersma.

Norway

Eivind Berge, Per Anton Sirnes, Erik Gjertsen, Torstein Hole, Knut Erga, Arne Hallaråker, Gunnar Skjelvan, Anders Østrem, Beraki Ghezai, Arne Svilaas, Peter Christersson, Torbjørn Øien, Svein Høegh Henrichsen, Jan Erik Otterstad, Jan Berg-Johansen.

Poland

Janina Stepinska, Andrzej Gieroba, Malgorzata Biedrzycka, Michal Ogorek, Beata Wozakowska-Kaplon, Krystyna Loboz-Grudzien, Jaroslaw, Wieslaw Supinski, Jerzy Kuzniar, Roman Zaluska, Jaroslaw Hiczkiewicz,

Lucyna Swiatkowska-Byczynska, Lech Kucharski, Marcin Gruchala, Piotr Minc, Maciej Olszewski, Grzegorz Kania, Malgorzata Krzciuk, Zbigniew Lajkowski, Bozenna Ostrowska-Pomian, Jerzy Lewczuk, Elzbieta Zinka, Agnieszka Karczmarczyk, Malgorzata Chmielnicka-Pruszczynska, Iwona Wozniak-Skowerska, Grzegorz Opolski, Marek Bronisz, Marcin Ogorek, Grazyna Glanowska, Piotr Ruszkowski, Grzegorz Skonieczny, Ryszard Sciborski, Boguslaw Okopien, Piotr Kukla, Krzysztof Galbas, Krzysztof Cymerman, Jaroslaw Jurowiecki, Pawel Miekus, Waldemar Myszka, Stanislaw Mazur, Roman Lysek, Jacek Baszak, Teresa Rusicka-Piekarz, Grzegorz Raczak, Ewa Domanska, Jadwiga Nessler, Jozef Lesnik.

Russia

Vera Eltishcheva, Roman Libis, Gadel Kamalov, Dmitry Belenky, Liudmila Egorova, Alexander Khokhlov, Eduard Yakupov, Dmitry Zateyshchikov, Olga Barbarash, Olga Miller, Evgeniy Mazur, Konstantin Zrazhevskiy, Tatyana Novikova, Yulia Moiseeva, Elena Polkanova, Konstantin Sobolev, Maria Rossovskaya, Yulia Shapovalova, Alla Kolesnikova, Konstantin Nikolaev, Oksana Zemlianskaia, Anna Zateyshchikova, Victor Kostenko, Sergey Popov, Maria Poltavskaya, Anton Edin,

Elena Aleksandrova, Oksana Drapkina, Alexander Vishnevsky, Oleg Nagibovich, Petr Chizhov, Svetlana Rachkova, Mikhail Sergeev, Borys Kurylo, Alexey Ushakov.

Spain

Xavier Vinolas, Pere Alvarez Garcia, Maria Fernanda Lopez Fernandez, Luis Tercedor Sanchez, Salvador Tranche Iparraguirre, Pere Toran Monserrat, Emilio Marquez Contreras, Jordi Isart Rafecas, Juan Motero Carrasco, Pablo Garcia Pavia, Casimiro Gomez Pajuelo, Luis Miguel Rincon Diaz, Luis Fernando Iglesias Alonso, Angel Grande Ruiz, Jordi Merce Klein, Jose Ramon Gonzalez Juanatey, Ines Monte Collado, Herminia Palacin Piquero, Carles Brotons Cuixart, Esther Fernandez Escobar, Joan Bayo i Llibre, Cecilia Corros Vicente, Manuel Vida Gutierrez, Francisco Epelde Gonzalo, Carlos Alexandre Almeida Fernandez, Encarnacion Martinez Navarro, Jordi Isart Rafecas, Juan Jose Montero Alia, Maria Barreda Gonzalez, Maria Angels Moleiro Oliva, Jose Iglesias Sanmartin, Mercedes Jimenez Gonzalez, Maria del Mar Rodriguez Alvarez, Juan Herreros Melenchon, Tomas Ripoll Vera, Manuel Jimenez Navarro, Maria Vazquez Caamano, Maria Fe Arcocha Torres, Gonzalo Marcos Gomez, Andres Iniguez Romo, Miguel Angel Prieto Diaz.

Sweden

Mårten Rosenqvist, Alexander Wirdby, Centrumkliniken, Jan Lindén, Kerstin Henriksson, Micael Elmersson, Arnor Egilsson, Ulf Börjesson, Gunnar Svärd, Bo Liu, Anders Lindh, Lars-Bertil Olsson, Mikael Gustavsson, Lars Andersson, Lars Benson, Claes Bothin, Ali Hajimirsadeghi, Björn Martinsson, Marianne Ericsson, Åke Ohlsson, Håkan Lindvall, Peter Svensson, Katarina Thörne, Hans Händel, Pyotr Platonov, Fredrik Bernsten, Ingar Timberg, Milita Crisby, Jan-Erik Karlsson, Agneta Andersson, Lennart Malmqvist, Johan Engdahl, Jörgen Thulin, Aida Hot-Bjelak, Steen Jensen, Per Stalby.

Switzerland

Jan Steffel, Johann Debrunner, Juerg H. Beer, Dipen Shah.

Ukraine

Iurii Rudyk, Vira Tseluyko, Oleksandr Karpenko, Svitlana Zhurba, Igor Kraiz, Oleksandr Parkhomenko, Iryna Kupnovytska, Nestor Seredyuk, Yuriy Mostovoy, Oleksiy Ushakov, Olena Koval, Igor Kovalskiy, Yevgeniya Svyshchenko, Oleg Sychov, Mykola Stanislavchuk, Andriy Yagensky, Susanna Tykhonova, Ivan Fushtey.

United Kingdom

Will Murdoch, Naresh Chauhan, Daryl Goodwin, Louise Lumley, Ramila Patel, Philip Saunders, Bennett Wong, Alex Cameron, Philip Saunders, Niranjan Patel, P Jhittay, Andrew Ross, M S Kainth, Karim Ladha, Kevin Douglas, Gill Pickavance, Joanna McDonnell, Laura Handscombe, Trevor Gooding, Helga Wagner, Cumberlidge, Colin Bradshaw, Catherine Bromham, Kevin Jones, Shoeb Suryani, Richard Coates, Bhupinder Sarai, W Willcock, S Sircar, John Cairns, A Gilliand, Roman Bilas, E Strieder, Peter Hutchinson, Anne Wakeman, Michael Stokes, Graham Kirby, Bhaskhar Vishwanathan, Nigel Bird, Paul Evans, M Clark, John Bisatt, Jennifer Litchfield, E Fisher, Tim Fooks, Richard Kelsall, Neil Paul, Elizabeth Alborough, Michael Aziz, C Ramesh, Pete Wilson, Simon Franklin, Sue Fairhead, Julian Thompson, Hasan Chowan, Gary Taylor, Dawn Tragen, Matt Parfitt, Claire Seamark, Carolyn Paul, Mark Richardson, Angus Jefferies, Helen Sharp, Hywel Jones, Claire Giles, Matthew Bramley, Philip Williams, Jehad Aldegather, Simon Wetherell, William Lumb, Phil Evans, Frances Scouller, Neil Macey, Stephen Rogers, Yvette Stipp, Richard West, Philip Pinney, Paul Wadeson, John Matthews, Preeti Pandya, Andrew Gallagher, T Railton, Emyr Davies, Jonathan McClure, Marc Jacobs, Claire Hutton, R Thompson, Bijoy Sinha, Keith Butter, Susan Barrow, Helen Little, David Russell, Ulka Choudhary, Ikram Haq,

Camm CF, *et al. Open Heart* 2022; 9:e002038. doi: 10.1136/openhrt-2022-002038

Paul Ainsworth, Claire Jones, Phil Weeks, Jane Eden, Lisa Gibbons, Janet Glencross, Alison MacLeod, K Poland, Conor Mulolland, A Warke, Paul Conn, D Burns, R Smith, R Kamath, Jonathan Webster, Ian Hodgins, Stephen Vercoe, Paul Roome, Hilary Pinnock, Jayesh Patel, Amar Ali, Nigel Hart, Richard Davies, Nigel De-Sousa, Catherine Neden, Mark Danielsen, Purnima Sharma, Sophia Galloway, Charlotte Hawkins, Raife Oliver, Martin Aylward, Mira Pattni, Gordon Irvine, Shahid Ahmad, Catherine Rothwell, Fiaz Choudhary, Sabrina Khalaque, Stephanie Short, Sharon Peters, Warwick Coulson, Neil Roberts,

Amy Butler, Steven Coates, Ben Ward, Daniel Jackson, Steve Walton, Diane Shepherd, Toh Wong, Mark Boon, Melanie Deacon, David Cornelius, Sarah Davies, Ben Frankel, Nick Hargreaves, Henry Choi, Jon Sumner, Tim Myhill, Salah Estifanos, Diane Geatch, Justin Wilkinson, Richard Veale, Karen Forshaw, Rob Hirst, Kashif Zaman, Catherine Liley, Rebecca Wastling, Paul McEleny, Andre Beattie, Philip Cooke, Mike Wong, Mark Pugsley, Chaminda Dooldeniya, Greg Rogers, James Bennett, Polly Jacobs, Rajesh Muvva, Matthew Adam, Robin Fox, Nicolas Thomas, Simon Cartwright, Rory Reed, Simon Randfield, Christine A'Court, Ann Flynn, Andrew Halpin, Shoeb Suryani, Simon Dobson, Louise Lomax, Minnal Nadaph, Iain Munro, Jane Goram, Helen Stoddart, Phil Simmons, John Shewring, Emma Bowen-Simpkins, Mark Rickenbach, Polly Jacobs.

Australia

Adam Blenkhorn, Bhuwanendu Singh, Penny Astridge, William van Gaal, Walter Abhayaratna, Philip Thomson, Ron Lehman, Jens Kilian, David Coulshed, Andrei Catanchin, David Colquhoun, Hosen Kiat, David Eccleston, John French, Bronte Ayres, Peter Blombery, Thanh Phan, James Rogers, David O'Donnell, Sang Cheol Bae, Harry Gibbs, Patrick Carroll, Greg Starmer, Margaret Arstall, Maurits Binnekamp, Astin Lee.

Canada

John Eikelboom, Robert Luton, Milan Gupta, Amritanshu Shekhar Pandey, Stephen Cheung, Rolland Leader, Philippe Beaudry, Félix Ayala-Paredes, Joseph Berlingieri, John Heath, Germain Poirier, Miranda du Preez, Bradley Schweitzer, Reginald Nadeau, Ripple Dhillon, Tomasz Hruczkowski, Andrea Lavoie, Ratika Parkash, James Cha, Benoit Coutu, Paul MacDonald, Brian Ramjattan, Jorge Bonet, Saul Vizel, Paul Angaran, Sameh Fikry.

Egypt

Camm CF, *et al. Open Heart* 2022; 9:e002038. doi: 10.1136/openhrt-2022-002038

Ahmed Mowafy, Azza Katta, Mazen Tawfik, Moustafa Nawar, Mohamed Sobhy, Seif Kamal Abou Seif, Tarek Khairy, Ahmed Abd El-Aziz, Nasser Taha, Ashraf Reda, Atef Elbahry, Mohamed Setiha, Mohamed Gamal El Din, Magdi Elkhadem, Adel El-Etreby.

South Africa

David Kettles, Junaid Bayat, Heidi Siebert, Adrian Horak, Ynez Kelfkens, Riaz Garda, Barry Jacobson, Thayabran Pillay, Michele Guerra, Louis van Zyl, Hendrik Theron, Andrew Murray, Rikus Louw, Deon Greyling, Pindile Mntla, Siddique Ismail, Fayzal Ahmed, Johannes Engelbrecht, Shambu Maharajh, Wessel Oosthuysen, Rehana Loghdey, Veronica Ueckermann.

United Arab Emirates

Wael Al Mahmeed, Abdullah Al Naeemi, Ghazi Yousef, Nooshin Bazargani, Munther AlOmairi, Rajan Maruthanayagam, Rupesh Singh, Ahmed Naguib, Mohamed Ibrahim, Amrish Agrawal, Mukesh Nathani, Ehab M. Esheiba, Adel Wassef, Rajeev Gupta.

United States

Michael Cox, Scott Beach, Peter Duffy, Stephen Falkowski, Kevin Ferrick, Miguel Franco, W. Michael Kutayli, Annette Quick, Niraj Sharma, Vance Wilson, Stephen Miller, Mark Alberts, Edwin Blumberg, Roddy Canosa, Ted Gutowski, Rodney Ison, Jorge Garcia, Paul Mullen, Howard Noveck, Pamela Rama, Rajneesh Reddy, Marcus Williams, Daniel Nishijima, Keith Ferdinand, Ihsan Haque, Robert Mendelson, Sridevi Pitta, Daniel Theodoro, Charles Treasure, Moustafa Moustafa, Cas Cader, Walter Pharr, Alisha Oropallo, George Platt, Jaspal Gujral, James Welker, Firas Koura.

GARFIELD-AF Ethics Committee List

Sponsor	Protocol #	Project Code	Region	Sub-Region	Country	Submission Requirement Type	Authority/Committee Name
Thrombosis Research Institute	TRI08888	IPAA4663	Latin America	South America	Argentina	CEC	Comite Independiente de Etica
Thrombosis Research Institute	TRI08888	IPAA4663	Latin America	South America	Argentina	CEC	Comite Independiente de Etica
Thrombosis Research Institute	TRI08888	IPAA4663	Latin America	South America	Argentina	CEC	Comite Independiente de Etica
Thrombosis Research Institute	TRI08888	IPAA4663	Latin America	South America	Argentina	CEC	Comite Independiente de Etica
Thrombosis Research Institute	TRI08888	IPAA4663	Latin America	South America	Argentina	RA	CCIS Comision Conjunta de Investigacion en Salud
Thrombosis Research Institute	TRI08888	IPAA4663	Latin America	South America	Argentina	CEC	Comite Independiente de Etica
Thrombosis Research Institute	TRI08888	IPAA4663	Latin America	South America	Argentina	RA	CCIS Comision Conjunta de Investigacion en Salud
Thrombosis Research Institute	TRI08888	IPAA4663	Latin America	South America	Argentina	RA	CCIS Comision Conjunta de Investigacion en Salud
Thrombosis Research Institute	TRI08888	IPAA4663	Latin America	South America	Argentina	RA	CCIS Comision Conjunta de Investigacion en Salud
Thrombosis Research Institute	TRI08888	IPAA4663	Latin America	South America	Argentina	CEC	Comite Independiente de Etica
Thrombosis Research Institute	TRI08888	IPAA4663	Latin America	South America	Argentina	Others	
Thrombosis Research Institute	TRI08888	IPAA4663	Asia Pacific	Australia & New Zealand	Australia	Central IRB	Metro South Health Service District Human Research Ethics Committee
Thrombosis Research Institute	TRI08888	IPAA4663	Asia Pacific	Australia & New Zealand	Australia	Central IRB	University of Wollongong & Illawarra HREC
Thrombosis Research Institute	TRI08888	IPAA4663	Asia Pacific	Australia & New Zealand	Australia	Central IRB	
Thrombosis Research Institute	TRI08888	IPAA4663	Asia Pacific	Australia & New Zealand	Australia	Central IRB	Metro South Health Service District Human Research Ethics Committee
Thrombosis Research Institute	TRI08888	IPAA4663	Europe/Middle East/Africa-EMEA	Eastern Europe/Middle East	Austria	CEC	Ethikkommission der Medizinischen Universität Graz

Camm CF, *et al. Open Heart* 2022; 9:e002038. doi: 10.1136/openhrt-2022-002038

Thrombosis Research Institute	TRI08888	IPAA4663	Europe/Middle East/Africa-EMEA	Western Europe	Belgium	CEC	Ethisch Comité UZA
Thrombosis Research Institute	TRI08888	IPAA4663	Europe/Middle East/Africa-EMEA	Western Europe	Belgium	CEC	Ethisch Comité UZA
Thrombosis Research Institute	TRI08888	IPAA4663	Europe/Middle East/Africa-EMEA	Western Europe	Belgium	CEC	Ethisch Comité UZA
Thrombosis Research Institute	TRI08888	IPAA4663	Europe/Middle East/Africa-EMEA	Western Europe	Belgium	CEC	Ethisch Comité UZA
Thrombosis Research Institute	TRI08888	IPAA4663	Europe/Middle East/Africa-EMEA	Western Europe	Belgium	CEC	Ethisch Comité UZA
Thrombosis Research Institute	TRI08888	IPAA4663	Europe/Middle East/Africa-EMEA	Western Europe	Belgium	CEC	Ethisch Comité UZA
Thrombosis Research Institute	TRI08888	IPAA4663	Europe/Middle East/Africa-EMEA	Western Europe	Belgium	CEC	Ethisch Comité UZA
Thrombosis Research Institute	TRI08888	IPAA4663	Europe/Middle East/Africa-EMEA	Western Europe	Belgium	CEC	Ethisch Comité UZA
Thrombosis Research Institute	TRI08888	IPAA4663	Europe/Middle East/Africa-EMEA	Western Europe	Belgium	CEC	Ethisch Comité UZA
Thrombosis Research Institute	TRI08888	IPAA4663	Europe/Middle East/Africa-EMEA	Western Europe	Belgium	CEC	Ethisch Comité UZA
Thrombosis Research Institute	TRI08888	IPAA4663	Latin America	South America	Brazil	CEC	
Thrombosis Research Institute	TRI08888	IPAA4663	Latin America	South America	Brazil	CEC	
Thrombosis Research Institute	TRI08888	IPAA4663	Latin America	South America	Brazil	CEC	
Thrombosis Research Institute	TRI08888	IPAA4663	USA/Canada	Canada	Canada	Central IRB	WIRB
Thrombosis Research Institute	TRI08888	IPAA4663	USA/Canada	Canada	Canada	Central IRB	WIRB
Thrombosis Research Institute	TRI08888	IPAA4663	USA/Canada	Canada	Canada	Central IRB	WIRB
Thrombosis Research Institute	TRI08888	IPAA4663	USA/Canada	Canada	Canada	Central IRB	WIRB

Thrombosis Research Institute	TRI08888	IPAA4663	Latin America	South America	Chile	RA	Instituto de Salud Pública de Chile
Thrombosis Research Institute	TRI08888	IPAA4663	Europe/Middle East/Africa-EMEA	Eastern Europe/Middle East	Czech Republic	RA	Statni ustav pro kontrolu leciv
Thrombosis Research Institute	TRI08888	IPAA4663	Europe/Middle East/Africa-EMEA	Eastern Europe/Middle East	Czech Republic	RA	Statni ustav pro kontrolu leciv
Thrombosis Research Institute	TRI08888	IPAA4663	Europe/Middle East/Africa-EMEA	Western Europe	Denmark	CEC	De Videnskabsetiske Komitéer for Region Hovedstaden
Thrombosis Research Institute	TRI08888	IPAA4663	Europe/Middle East/Africa-EMEA	Western Europe	Denmark	CEC	De Videnskabsetiske Komitéer for Region Hovedstaden
Thrombosis Research Institute	TRI08888	IPAA4663	Europe/Middle East/Africa-EMEA	Western Europe	Denmark	CEC	De Videnskabsetiske Komitéer for Region Hovedstaden
Thrombosis Research Institute	TRI08888	IPAA4663	Europe/Middle East/Africa-EMEA	Western Europe	Denmark	CEC	De Videnskabsetiske Komitéer for Region Hovedstaden
Thrombosis Research Institute	TRI08888	IPAA4663	Europe/Middle East/Africa-EMEA	Western Europe	Denmark	CEC	De Videnskabsetiske Komitéer for Region Hovedstaden
Thrombosis Research Institute	TRI08888	IPAA4663	Europe/Middle East/Africa-EMEA	Western Europe	Denmark	CEC	De Videnskabsetiske Komitéer for Region Hovedstaden
Thrombosis Research Institute	TRI08888	IPAA4663	Europe/Middle East/Africa-EMEA	Western Europe	Finland	CEC	Pirkanmaan sairaanhoitopiirin eettinen toimikunta
Thrombosis Research Institute	TRI08888	IPAA4663	Europe/Middle East/Africa-EMEA	Western Europe	Finland	CEC	Pirkanmaan sairaanhoitopiirin eettinen toimikunta
Thrombosis Research Institute	TRI08888	IPAA4663	Europe/Middle East/Africa-EMEA	Western Europe	Finland	CEC	Pirkanmaan sairaanhoitopiirin eettinen toimikunta
Thrombosis Research Institute	TRI08888	IPAA4663	Europe/Middle East/Africa-EMEA	Western Europe	Finland	CEC	Pirkanmaan sairaanhoitopiirin eettinen toimikunta
Thrombosis Research Institute	TRI08888	IPAA4663	Europe/Middle East/Africa-EMEA	Western Europe	France	CEC	Comité Consultatif sur le Traitement de l'information Recherche/Santé
Thrombosis Research Institute	TRI08888	IPAA4663	Europe/Middle East/Africa-EMEA	Western Europe	France	CEC	Comité Consultatif sur le Traitement de l'information Recherche/Santé

Camm CF, *et al. Open Heart* 2022; 9:e002038. doi: 10.1136/openhrt-2022-002038

Thrombosis Research Institute	TRI08888	IPAA4663	Europe/Middle East/Africa-EMEA	Western Europe	France	CEC	Comité Consultatif sur le Traitement de l'information Recherche/Santé
Thrombosis Research Institute	TRI08888	IPAA4663	Europe/Middle East/Africa-EMEA	Western Europe	France	CEC	Comité Consultatif sur le Traitement de l'information Recherche/Santé
Thrombosis Research Institute	TRI08888	IPAA4663	Europe/Middle East/Africa-EMEA	Western Europe	France	CEC	Comité Consultatif sur le Traitement de l'information Recherche/Santé
Thrombosis Research Institute	TRI08888	IPAA4663	Europe/Middle East/Africa-EMEA	Western Europe	France	CEC	Comité Consultatif sur le Traitement de l'information Recherche/Santé
Thrombosis Research Institute	TRI08888	IPAA4663	Europe/Middle East/Africa-EMEA	Western Europe	France	CEC	Comité Consultatif sur le Traitement de l'information Recherche/Santé
Thrombosis Research Institute	TRI08888	IPAA4663	Europe/Middle East/Africa-EMEA	Western Europe	France	CEC	Comité Consultatif sur le Traitement de l'information Recherche/Santé
Thrombosis Research Institute	TRI08888	IPAA4663	Europe/Middle East/Africa-EMEA	Eastern Europe/Middle East	Hungary	CEC	Egeszsegugyi Tudomanyos Tanacs Tudomanyos es Kutatasetikai Bizottsag
Thrombosis Research Institute	TRI08888	IPAA4663	Europe/Middle East/Africa-EMEA	Eastern Europe/Middle East	Hungary	CEC	Egeszsegugyi Tudomanyos Tanacs Tudomanyos es Kutatasetikai Bizottsag
Thrombosis Research Institute	TRI08888	IPAA4663	Europe/Middle East/Africa-EMEA	Eastern Europe/Middle East	Hungary	RA	Orszagos Gyogyszereszteti es Elelmezes-egszsegugyi Intezet
Thrombosis Research Institute	TRI08888	IPAA4663	Europe/Middle East/Africa-EMEA	Eastern Europe/Middle East	Hungary	CEC	Egeszsegugyi Tudomanyos Tanacs Tudomanyos es Kutatasetikai Bizottsag
Thrombosis Research Institute	TRI08888	IPAA4663	Europe/Middle East/Africa-EMEA	Eastern Europe/Middle East	Hungary	RA	Orszagos Gyogyszereszteti es Elelmezes-egszsegugyi Intezet
Thrombosis Research Institute	TRI08888	IPAA4663	Europe/Middle East/Africa-EMEA	Eastern Europe/Middle East	Hungary	CEC	Egeszsegugyi Tudomanyos Tanacs Tudomanyos es Kutatasetikai Bizottsag
Thrombosis Research Institute	TRI08888	IPAA4663	Europe/Middle East/Africa-EMEA	Eastern Europe/Middle East	Hungary	RA	Orszagos Gyogyszereszteti es Elelmezes-egszsegugyi Intezet
Thrombosis Research Institute	TRI08888	IPAA4663	Europe/Middle East/Africa-EMEA	Eastern Europe/Middle East	Hungary	CEC	Egeszsegugyi Tudomanyos Tanacs Tudomanyos es Kutatasetikai Bizottsag

Thrombosis Research Institute	TRI08888	IPAA4663	Europe/Middle East/Africa-EMEA	Eastern Europe/Middle East	Hungary	RA	Orszagos Gyogyszereszteti es Elelmezes-egszsegugyi Intezet
Thrombosis Research Institute	TRI08888	IPAA4663	Europe/Middle East/Africa-EMEA	Eastern Europe/Middle East	Hungary	RA	Orszagos Gyogyszereszteti es Elelmezes-egszsegugyi Intezet
Thrombosis Research Institute	TRI08888	IPAA4663	Europe/Middle East/Africa-EMEA	Eastern Europe/Middle East	Hungary	RA	Orszagos Gyogyszereszteti es Elelmezes-egszsegugyi Intezet
Thrombosis Research Institute	TRI08888	IPAA4663	Europe/Middle East/Africa-EMEA	Eastern Europe/Middle East	Hungary	CEC	Egeszsegugyi Tudomanyos Tanacs Tudomanyos es Kutatasetikai Bizottsag
Thrombosis Research Institute	TRI08888	IPAA4663	Europe/Middle East/Africa-EMEA	Eastern Europe/Middle East	Hungary	CEC	Egeszsegugyi Tudomanyos Tanacs Tudomanyos es Kutatasetikai Bizottsag
Thrombosis Research Institute	TRI08888	IPAA4663	Europe/Middle East/Africa-EMEA	Eastern Europe/Middle East	Hungary	CEC	Egeszsegugyi Tudomanyos Tanacs Tudomanyos es Kutatasetikai Bizottsag
Thrombosis Research Institute	TRI08888	IPAA4663	Europe/Middle East/Africa-EMEA	Eastern Europe/Middle East	Hungary	RA	Orszagos Gyogyszereszteti es Elelmezes-egszsegugyi Intezet
Thrombosis Research Institute	TRI08888	IPAA4663	Europe/Middle East/Africa-EMEA	Eastern Europe/Middle East	Hungary	RA	Orszagos Gyogyszereszteti es Elelmezes-egszsegugyi Intezet
Thrombosis Research Institute	TRI08888	IPAA4663	Europe/Middle East/Africa-EMEA	Eastern Europe/Middle East	Hungary	RA	Orszagos Gyogyszereszteti es Elelmezes-egszsegugyi Intezet
Thrombosis Research Institute	TRI08888	IPAA4663	Europe/Middle East/Africa-EMEA	Eastern Europe/Middle East	Hungary	RA	Orszagos Gyogyszereszteti es Elelmezes-egszsegugyi Intezet
Thrombosis Research Institute	TRI08888	IPAA4663	Europe/Middle East/Africa-EMEA	Eastern Europe/Middle East	Hungary	RA	
Thrombosis Research Institute	TRI08888	IPAA4663	Asia Pacific	India Region	India	RA	Directorate General of Health Services (India)
Thrombosis Research Institute	TRI08888	IPAA4663	Asia Pacific	India Region	India	RA	Directorate General of Health Services (India)
Thrombosis Research Institute	TRI08888	IPAA4663	Europe/Middle East/Africa-EMEA	Western Europe	Italy	CEC	Comitato Etico delle Aziende Sanitarie dell'Umbria di Perugia
Thrombosis Research Institute	TRI08888	IPAA4663	Japan	Japan	Japan	RA	

Thrombosis Research Institute	TRI08888	IPAA4663	Japan	Japan	Japan	RA	
Thrombosis Research Institute	TRI08888	IPAA4663	Asia Pacific	Southeast Asia	Korea, Republic of	RA	
Thrombosis Research Institute	TRI08888	IPAA4663	Latin America	Central America	Mexico	RA	COFEPRIS - Comision de Autorizacion Sanitaria (Mexico)
Thrombosis Research Institute	TRI08888	IPAA4663	Latin America	Central America	Mexico	RA	COFEPRIS - Comision de Autorizacion Sanitaria (Mexico)
Thrombosis Research Institute	TRI08888	IPAA4663	Latin America	Central America	Mexico	RA	COFEPRIS - Comision de Autorizacion Sanitaria (Mexico)
Thrombosis Research Institute	TRI08888	IPAA4663	Latin America	Central America	Mexico	RA	COFEPRIS - Comision de Autorizacion Sanitaria (Mexico)
Thrombosis Research Institute	TRI08888	IPAA4663	Latin America	Central America	Mexico	RA	COFEPRIS - Comision de Autorizacion Sanitaria (Mexico)
Thrombosis Research Institute	TRI08888	IPAA4663	Latin America	Central America	Mexico	RA	COFEPRIS - Comision de Autorizacion Sanitaria (Mexico)
Thrombosis Research Institute	TRI08888	IPAA4663	Latin America	Central America	Mexico	RA	COFEPRIS - Comision de Autorizacion Sanitaria (Mexico)
Thrombosis Research Institute	TRI08888	IPAA4663	Latin America	Central America	Mexico	RA	COFEPRIS - Comision de Autorizacion Sanitaria (Mexico)
Thrombosis Research Institute	TRI08888	IPAA4663	Latin America	Central America	Mexico	RA	COFEPRIS - Comision de Autorizacion Sanitaria (Mexico)
Thrombosis Research Institute	TRI08888	IPAA4663	Latin America	Central America	Mexico	RA	COFEPRIS - Comision de Autorizacion Sanitaria (Mexico)
Thrombosis Research Institute	TRI08888	IPAA4663	Latin America	Central America	Mexico	RA	COFEPRIS - Comision de Autorizacion Sanitaria (Mexico)
Thrombosis Research Institute	TRI08888	IPAA4663	Latin America	Central America	Mexico	RA	COFEPRIS - Comision de Autorizacion Sanitaria (Mexico)
Thrombosis Research Institute	TRI08888	IPAA4663	Latin America	Central America	Mexico	RA	COFEPRIS - Comision de Autorizacion Sanitaria (Mexico)
Thrombosis Research Institute	TRI08888	IPAA4663	Latin America	Central America	Mexico	RA	COFEPRIS - Comision de Autorizacion Sanitaria (Mexico)
Thrombosis Research Institute	TRI08888	IPAA4663	Latin America	Central America	Mexico	RA	COFEPRIS - Comision de Autorizacion Sanitaria (Mexico)
Thrombosis Research Institute	TRI08888	IPAA4663	Latin America	Central America	Mexico	RA	COFEPRIS - Comision de Autorizacion Sanitaria (Mexico)
Thrombosis Research Institute	TRI08888	IPAA4663	Latin America	Central America	Mexico	RA	COFEPRIS - Comision de Autorizacion Sanitaria (Mexico)
Thrombosis Research Institute	TRI08888	IPAA4663	Latin America	Central America	Mexico	RA	COFEPRIS - Comision de Autorizacion Sanitaria (Mexico)

Thrombosis Research Institute	TRI08888	IPAA4663	Latin America	Central America	Mexico	RA	COFEPRIS - Comision de Autorizacion Sanitaria (Mexico)
Thrombosis Research Institute	TRI08888	IPAA4663	Latin America	Central America	Mexico	RA	COFEPRIS - Comision de Autorizacion Sanitaria (Mexico)
Thrombosis Research Institute	TRI08888	IPAA4663	Latin America	Central America	Mexico	RA	COFEPRIS - Comision de Autorizacion Sanitaria (Mexico)
Thrombosis Research Institute	TRI08888	IPAA4663	Latin America	Central America	Mexico	RA	COFEPRIS - Comision de Autorizacion Sanitaria (Mexico)
Thrombosis Research Institute	TRI08888	IPAA4663	Latin America	Central America	Mexico	RA	COFEPRIS - Comision de Autorizacion Sanitaria (Mexico)
Thrombosis Research Institute	TRI08888	IPAA4663	Latin America	Central America	Mexico	RA	COFEPRIS - Comision de Autorizacion Sanitaria (Mexico)
Thrombosis Research Institute	TRI08888	IPAA4663	Latin America	Central America	Mexico	RA	COFEPRIS - Comision de Autorizacion Sanitaria (Mexico)
Thrombosis Research Institute	TRI08888	IPAA4663	Latin America	Central America	Mexico	RA	COFEPRIS - Comision de Autorizacion Sanitaria (Mexico)
Thrombosis Research Institute	TRI08888	IPAA4663	Latin America	Central America	Mexico	RA	COFEPRIS - Comision de Autorizacion Sanitaria (Mexico)
Thrombosis Research Institute	TRI08888	IPAA4663	Latin America	Central America	Mexico	RA	COFEPRIS - Comision de Autorizacion Sanitaria (Mexico)
Thrombosis Research Institute	TRI08888	IPAA4663	Latin America	Central America	Mexico	RA	COFEPRIS - Comision de Autorizacion Sanitaria (Mexico)
Thrombosis Research Institute	TRI08888	IPAA4663	Latin America	Central America	Mexico	RA	COFEPRIS - Comision de Autorizacion Sanitaria (Mexico)
Thrombosis Research Institute	TRI08888	IPAA4663	Latin America	Central America	Mexico	RA	COFEPRIS - Comision de Autorizacion Sanitaria (Mexico)
Thrombosis Research Institute	TRI08888	IPAA4663	Latin America	Central America	Mexico	RA	COFEPRIS - Comision de Autorizacion Sanitaria (Mexico)
Thrombosis Research Institute	TRI08888	IPAA4663	Latin America	Central America	Mexico	RA	COFEPRIS - Comision de Autorizacion Sanitaria (Mexico)
Thrombosis Research Institute	TRI08888	IPAA4663	Latin America	Central America	Mexico	RA	COFEPRIS - Comision de Autorizacion Sanitaria (Mexico)
Thrombosis Research Institute	TRI08888	IPAA4663	Latin America	Central America	Mexico	RA	COFEPRIS - Comision de Autorizacion Sanitaria (Mexico)
Thrombosis Research Institute	TRI08888	IPAA4663	Latin America	Central America	Mexico	RA	COFEPRIS - Comision de Autorizacion Sanitaria (Mexico)
Thrombosis Research Institute	TRI08888	IPAA4663	Latin America	Central America	Mexico	RA	COFEPRIS - Comision de Autorizacion Sanitaria (Mexico)
Thrombosis Research Institute	TRI08888	IPAA4663	Latin America	Central America	Mexico	RA	COFEPRIS - Comision de Autorizacion Sanitaria (Mexico)

Thrombosis Research Institute	TRI08888	IPAA4663	Latin America	Central America	Mexico	RA	COFEPRIS - Comision de Autorizacion Sanitaria (Mexico)
Thrombosis Research Institute	TRI08888	IPAA4663	Latin America	Central America	Mexico	RA	COFEPRIS - Comision de Autorizacion Sanitaria (Mexico)
Thrombosis Research Institute	TRI08888	IPAA4663	Latin America	Central America	Mexico	RA	COFEPRIS - Comision de Autorizacion Sanitaria (Mexico)
Thrombosis Research Institute	TRI08888	IPAA4663	Latin America	Central America	Mexico	RA	COFEPRIS - Comision de Autorizacion Sanitaria (Mexico)
Thrombosis Research Institute	TRI08888	IPAA4663	Latin America	Central America	Mexico	RA	COFEPRIS - Comision de Autorizacion Sanitaria (Mexico)
Thrombosis Research Institute	TRI08888	IPAA4663	Latin America	Central America	Mexico	RA	COFEPRIS - Comision de Autorizacion Sanitaria (Mexico)
Thrombosis Research Institute	TRI08888	IPAA4663	Latin America	Central America	Mexico	RA	COFEPRIS - Comision de Autorizacion Sanitaria (Mexico)
Thrombosis Research Institute	TRI08888	IPAA4663	Latin America	Central America	Mexico	RA	COFEPRIS - Comision de Autorizacion Sanitaria (Mexico)
Thrombosis Research Institute	TRI08888	IPAA4663	Europe/Middle East/Africa-EMEA	Western Europe	Netherlands	CEC	
Thrombosis Research Institute	TRI08888	IPAA4663	Europe/Middle East/Africa-EMEA	Western Europe	Norway	CEC	REK Sør-øst
Thrombosis Research Institute	TRI08888	IPAA4663	Europe/Middle East/Africa-EMEA	Western Europe	Norway	CEC	REK Sør-øst
Thrombosis Research Institute	TRI08888	IPAA4663	Europe/Middle East/Africa-EMEA	Eastern Europe/Middle East	Poland	CEC	Terenowa Komisja Bioetyczna przy Instytucie Kardiologii
Thrombosis Research Institute	TRI08888	IPAA4663	Europe/Middle East/Africa-EMEA	Eastern Europe/Middle East	Poland	CEC	Terenowa Komisja Bioetyczna przy Instytucie Kardiologii
Thrombosis Research Institute	TRI08888	IPAA4663	Europe/Middle East/Africa-EMEA	Eastern Europe/Middle East	Poland	CEC	Terenowa Komisja Bioetyczna przy Instytucie Kardiologii
Thrombosis Research Institute	TRI08888	IPAA4663	Europe/Middle East/Africa-EMEA	Eastern Europe/Middle East	Poland	CEC	Terenowa Komisja Bioetyczna przy Instytucie Kardiologii
Thrombosis Research Institute	TRI08888	IPAA4663	Europe/Middle East/Africa-EMEA	Eastern Europe/Middle East	Poland	CEC	Terenowa Komisja Bioetyczna przy Instytucie Kardiologii
Thrombosis Research Institute	TRI08888	IPAA4663	Europe/Middle East/Africa-EMEA	Eastern Europe/Middle East	Poland	CEC	Terenowa Komisja Bioetyczna przy Instytucie Kardiologii

Thrombosis Research Institute	TRI08888	IPAA4663	Europe/Middle East/Africa-EMEA	Eastern Europe/Middle East	Poland	CEC	Terenowa Komisja Bioetyczna przy Instytucie Kardiologii
Thrombosis Research Institute	TRI08888	IPAA4663	Europe/Middle East/Africa-EMEA	Eastern Europe/Middle East	Russian Federation	CEC	IIC of Ethic Assessment of Clinical Researches
Thrombosis Research Institute	TRI08888	IPAA4663	Europe/Middle East/Africa-EMEA	Eastern Europe/Middle East	Russian Federation	CEC	IIC of Ethic Assessment of Clinical Researches
Thrombosis Research Institute	TRI08888	IPAA4663	Europe/Middle East/Africa-EMEA	Eastern Europe/Middle East	Russian Federation	CEC	IIC of Ethic Assessment of Clinical Researches
Thrombosis Research Institute	TRI08888	IPAA4663	Europe/Middle East/Africa-EMEA	Eastern Europe/Middle East	Russian Federation	CEC	IIC of Ethic Assessment of Clinical Researches
Thrombosis Research Institute	TRI08888	IPAA4663	Europe/Middle East/Africa-EMEA	Eastern Europe/Middle East	Russian Federation	CEC	IIC of Ethic Assessment of Clinical Researches
Thrombosis Research Institute	TRI08888	IPAA4663	Europe/Middle East/Africa-EMEA	Eastern Europe/Middle East	Russian Federation	CEC	IIC of Ethic Assessment of Clinical Researches
Thrombosis Research Institute	TRI08888	IPAA4663	Europe/Middle East/Africa-EMEA	Eastern Europe/Middle East	Russian Federation	CEC	IIC of Ethic Assessment of Clinical Researches
Thrombosis Research Institute	TRI08888	IPAA4663	Europe/Middle East/Africa-EMEA	Eastern Europe/Middle East	Russian Federation	CEC	IIC of Ethic Assessment of Clinical Researches
Thrombosis Research Institute	TRI08888	IPAA4663	Asia Pacific	Southeast Asia	Singapore	RA	
Thrombosis Research Institute	TRI08888	IPAA4663	Europe/Middle East/Africa-EMEA	Africa	South Africa	RA	MCC - Medicines Control Council (South Africa)
Thrombosis Research Institute	TRI08888	IPAA4663	Europe/Middle East/Africa-EMEA	Africa	South Africa	RA	MCC - Medicines Control Council (South Africa)
Thrombosis Research Institute	TRI08888	IPAA4663	Europe/Middle East/Africa-EMEA	Africa	South Africa	RA	MCC - Medicines Control Council (South Africa)
Thrombosis Research Institute	TRI08888	IPAA4663	Europe/Middle East/Africa-EMEA	Western Europe	Spain	CEC	CEIC Hospital Universitario Virgen de la Victoria
Thrombosis Research Institute	TRI08888	IPAA4663	Europe/Middle East/Africa-EMEA	Western Europe	Spain	CEC	CEIC Hospital Universitario Virgen de la Victoria

Camm CF, *et al. Open Heart* 2022; 9:e002038. doi: 10.1136/openhrt-2022-002038

Thrombosis Research Institute	TRI08888	IPAA4663	Europe/Middle East/Africa-EMEA	Western Europe	Sweden	CEC	Regionala etikprövningsnämnden i Stockholm
Thrombosis Research Institute	TRI08888	IPAA4663	Europe/Middle East/Africa-EMEA	Western Europe	Sweden	CEC	Regionala etikprövningsnämnden i Stockholm
Thrombosis Research Institute	TRI08888	IPAA4663	Europe/Middle East/Africa-EMEA	Western Europe	Sweden	CEC	Regionala etikprövningsnämnden i Stockholm
Thrombosis Research Institute	TRI08888	IPAA4663	Europe/Middle East/Africa-EMEA	Western Europe	Sweden	CEC	Regionala etikprövningsnämnden i Stockholm
Thrombosis Research Institute	TRI08888	IPAA4663	Europe/Middle East/Africa-EMEA	Western Europe	Sweden	CEC	Regionala etikprövningsnämnden i Stockholm
Thrombosis Research Institute	TRI08888	IPAA4663	Europe/Middle East/Africa-EMEA	Western Europe	Sweden	CEC	Regionala etikprövningsnämnden i Stockholm
Thrombosis Research Institute	TRI08888	IPAA4663	Europe/Middle East/Africa-EMEA	Western Europe	Sweden	CEC	Regionala etikprövningsnämnden i Stockholm
Thrombosis Research Institute	TRI08888	IPAA4663	Europe/Middle East/Africa-EMEA	Western Europe	Sweden	CEC	Regionala etikprövningsnämnden i Stockholm
Thrombosis Research Institute	TRI08888	IPAA4663	Europe/Middle East/Africa-EMEA	Western Europe	Sweden	CEC	Regionala etikprövningsnämnden i Stockholm
Thrombosis Research Institute	TRI08888	IPAA4663	Europe/Middle East/Africa-EMEA	Western Europe	Sweden	CEC	Regionala etikprövningsnämnden i Stockholm
Thrombosis Research Institute	TRI08888	IPAA4663	Europe/Middle East/Africa-EMEA	Western Europe	Sweden	CEC	Regionala etikprövningsnämnden i Stockholm
Thrombosis Research Institute	TRI08888	IPAA4663	Europe/Middle East/Africa-EMEA	Western Europe	Sweden	CEC	Regionala etikprövningsnämnden i Stockholm
Thrombosis Research Institute	TRI08888	IPAA4663	Europe/Middle East/Africa-EMEA	Western Europe	Sweden	CEC	Regionala etikprövningsnämnden i Stockholm
Thrombosis Research Institute	TRI08888	IPAA4663	Europe/Middle East/Africa-EMEA	Western Europe	Switzerland	RA	
Thrombosis Research Institute	TRI08888	IPAA4663	Asia Pacific	Southeast Asia	Thailand	RA	

Thrombosis Research Institute	TRI08888	IPAA4663	Europe/Middle East/Africa-EMEA	Eastern Europe/Middle East	Turkey	CEC	Malatya Klinik Arastirmalar Etik Kurulu
Thrombosis Research Institute	TRI08888	IPAA4663	Europe/Middle East/Africa-EMEA	Eastern Europe/Middle East	Turkey	RA	Turkish Ministry of Health
Thrombosis Research Institute	TRI08888	IPAA4663	Europe/Middle East/Africa-EMEA	Eastern Europe/Middle East	Turkey	CEC	Malatya Klinik Arastirmalar Etik Kurulu
Thrombosis Research Institute	TRI08888	IPAA4663	Europe/Middle East/Africa-EMEA	Eastern Europe/Middle East	Ukraine	CEC	Central Ethics Commission of the Ministry of Health of Ukraine

Camm CF, *et al. Open Heart* 2022; 9:e002038. doi: 10.1136/openhrt-2022-002038
